# Flexible four-port MIMO antenna loaded with frequency selective surface for on-body applications

**DOI:** 10.1038/s41598-025-22301-x

**Published:** 2025-11-03

**Authors:** Manish Sharma, Dinesh Kumar Singh, Kanhaiya Sharma, Shashank Awasthi, Rana Gill, Tanweer Ali

**Affiliations:** 1 Chitkara University Institute of Engineering and Technology, Chitkara University, Rajpura, Punjab India; 2 Electronics and Communication Engineering Department, GL Bajaj Institute of Technology and Management, Greater Noida, UP India; 3 Department of Computer Science and Engineering, Symbiosis Institute of Technology, Symbiosis International (Deemed University), Pune, India; 4 Computer Science and Engineering Department, GL Bajaj Institute of Technology and Management, Greater Noida, UP India; 5 University Centre for Research and Development Department of Electronics and Communication Engineering, Chandigarh University, Gharuan, Mohali, Punjab India; 6 Department of Electronics and Communication Engineering, Manipal Institute of Technology, Manipal Academy of Higher Education, Manipal, 576104 India

**Keywords:** MIMO, CMA_DWA_, FSS_DWA_-array, SAR_DWA_, Conformal, ECC_DWA_, DG_DWA_, TARC_DWA_, CCL_DWA_, MEG_DWA_, Energy science and technology, Engineering

## Abstract

This work reports a dual-band four-port MIMO antenna loaded with a 5 x 5 frequency-selective-surface (FSS) of size 72.5 mm×75.0 mm for gain enhancement. The antenna uses a novel radiating-patch and a modified rectangular-ground printed on a 0.254 mm thickness Rogers substrate, generating bandwidths of 4.04–6.64 GHz and 7.44–16.60 GHz with overall dimensions of 30.0 mm×30.0 mm. The FSS, which is printed on FR4 1.60 mm substrate, is placed below the antenna at a distance of 15.0 mm, which records a maximum peak-gain of 10.77 dBi. The characteristics-mode-analysis _DWA_ (CMA_DWA_) is simulated by subjecting the antenna to 10 modes, with Mode-2, Mode-4, Mode-5, Mode-6, Mode-7, and Mode-9 being the significant modes with modal significance values more than 0.707 and generating six resonance values at 6.67 GHz, 7.20 GHz, 7.958 GHz, 8.76 GHz, 9.20 GHz, and 11.80 GHz. The four identical radiating patches are arranged in orthogonal sequence, achieving spatial-diversity performance with ECC_DWA_ ≤ 0.07 (Band-A), 0.03 (Band-B), DG_DWA_≥ 9.70dB (Band-A), 9.85dB (Band-B), TARC_DWA_≤ -5.0dB (Band-A), -2.50dB (Band-B), CCL_DWA_≤ 0.38 b/s/Hz (Band-A), 0.30 b/s/Hz (Band-B), and the difference between the MEG_DWA_ of two-ports to be $\cong$0.0dB. The SAR_DWA_ value corresponds to 0.158 W/Kg at 5.50 GHz, 0.076 W/Kg at 5.90 GHz, 0.0503 W/kg at 7.50 GHz, and 0.24 W/Kg at 10.0 GHz with conformal angles of 15^o^, 30^o^, and 45^0^, retaining the operational bandwidth.

## Introduction

The development of wireless technology has witnessed a faster data rate of transmission in the last few decades due to the evolution of the microstrip patch antenna, serving several applications. Also, the integrated multiple-input-multiple-output (MIMO_DWA_) technology in a single-port antenna configuration achieves desirable diversity schemes, enhancing the operational bandwidth with increased efficiency. The need for MIMO configuration is desirable for faster data rate transfer with high gain, which can be achieved by using a frequency-selective surface (FSS_DWA_). Also, the conformal antenna can be useful for flexible electronics applications. This concludes the requirement of MIMO_MBA_ antenna with multi-band applications, achieving high gain and flexible configuration for on-body applications, with analysis of SAR at selected frequency values.

The two radiating patches placed adjacent to each other^1^ achieve isolation of more than 23.0dB by using the neutralization line technique, which cancels the current vector flowing in the opposite direction. The MIMO antenna achieves an operational − 10.0 dB bandwidth of 3.52–10.08 GHz. The ultra-wideband MIMO antenna with bandwidth ranging between 3.30 and 13.84 GHz utilizes meta-material structure, and the placing of four identical patches in orthogonal sequence on the micro-machined substrate achieves isolation of more than 14.0dB^2–5^. The other analysis of having insightful analysis for a four-port orthogonal MIMO antenna is by subjecting the proposed work to characteristics-mode-analysis (CMA) and exciting the antenna by 10 modes with identifying the most significant mode, which decides the operational bandwidth with modal-significance values more than 0.707^6^. The reconfigurable configuration of the notched-band UWB-MIMO antenna is achieved by using PIN diodes^7^, and the antenna achieves isolation of more than 20.0 dB by using 45◦ placed T-shaped stubs in between them. Fractal-four-port MIMO antenna with a wavelength dimension of 0.46λo×0.46λo (λo = 2.08 GHz) uses a reflector to improve the matching of the impedance^8^, and the antenna configuration is also realized by using an equivalent-circuit model. A simple rectangular patch antenna with multiple slotted partial-ground and connected L-shaped strips in the ground^9–10^ achieves a UWB configuration with a maximum peak gain of 5.82 dBi. The Koch-fractal MIMO antenna achieves a maximum gain of 1.20 dBi with an efficiency of more than 80% in the IoT and sub-6.0 GHz band^11^. The combination of the half-cut semicircular slot with stepped microstrip feed achieves − 10.0dB bandwidth of 3.10–13.10 GHz^12^ with orthogonal four-port MIMO-antenna achieving dimension of 45 mm×45 mm MIMO antenna with 1 × 4 configuration applicable for n79-5G band utilizes Rogers substrate with thickness of 0.50 mm^13–16^ dual-band antenna with CPW-feed is transformed to MIMO-configuration by interconnecting ground and placing the patch orthogonally with a slit attached to ground for better isolation^17^, and a multi-band 3-D MIMO antenna placed in two-planes achieves horizontal-vertical polarization^18^. The combination of an orthogonal-shaped split-ring resonator designed as a meta-material and CPW-feed forms a two-port MIMO configuration^19–22^, which is separated by a decoupling structure attached to the ground and is useful for UWB applications. Four-radiating patch with a rectangular shape achieves resonance at 60.0 GHz, where the quarter-wave transformer is used for matching the impedance between the 50Ω microstrip and the patch^23,24^. A dual-band four-port MIMO antenna designed for 28.0 GHz and 38.0 GHz is formed by placing two identical patches adjacent and the other pair in a mirrored image sequence to achieve isolation of more than 20.0 dB^25–29^. Two-element array arranged in a four-port MIMO configuration in orthogonal-sequence uses a split-ring resonator as a filter with a patch achieving a UWB bandwidth^30–33^. Two-port antenna with a modified half-elliptical ground achieves a super-wideband configuration with a slotted funnel-shaped stub attached to the ground to achieve higher isolation^34^. Tri-band four-port MIMO antenna also supported by an equivalent circuit model^35^ uses a self-isolation technique to achieve better isolation with a pair of parasitic elements placed in between the gap of two radiating patches^35–37^, Transmission Coefficient^38^. The back-lobe radiation in a two-port MIMO antenna is reduced by placing repetitive structures below the antenna, which reduces the specific absorption rate^39, 40^. The gain of the antenna is increased by using an artificial magnetic conductor placed below the antenna, which increases the gain^41^, and a four-port MIMO antenna with orthogonal orientation, including an etched slit to resonate for 28.0 GHz/38.0 GHz application, with a parasitic structure placed between them for higher isolation. Two-port MIMO antenna with an overall dimension of 48 × 80 mm^2^ achieving isolation of more than 30.0dB, and a dual dual-band metamaterial is used as an absorber that reduces SAR^42^. Also, felt-substrate with flexible capability is used, generating three narrow bands which also integrate a flexible meta-surface to reduce backward radiation^43^. Single-port modified circular-patch antenna loaded with FSS achieves UWB-bandwidth with maximum peak-gain of 5.0 dB at 9.0 GHZ^44^, and a four-port MIMO antenna with ring-structured-patch and defected-ground generates sub-6.0 GHz bandwidth of 3.50 GHz-6.0 GHZ with gain enhancement achieved by using 5 × 5 FSS-array structure^45^. Also, FSS integrated a two-port MIMO antenna with a Chaired-shape structure observes a peak-gain of 7.96dBi which also observes high isolation^46^, and a dual-band four-port MIMO antenna targeting sub-6.0 GHz & X-band uses a Vivaldi-shape modified patch is also loaded with a split-ring MTM structure, which improves gain^47^.

This work discusses the integrated four-port MIMO_DWA_ dual-wideband of area 900 mm^2^ with novel FSS_DWA_ of size 72.50 × 75.0 mm^2^. The proposed antenna is printed on either surface of Rogers-dielectric material with a thickness of 0.254 mm, and FSS_DWA_ is printed on one surface of FR4 substrate with a thickness of 1.60 mm. The integration of the antenna with FSS_MBA_ enhances the peak-realized gain of the antenna. The proposed antenna with FSS_MBA_ includes the following characteristics.

- Four-port MIMO_DWA_ dual-wideband with a bandwidth of 4.04–6.64 GHz and 7.44–16.60 GHz is applicable for multi-bands, including WLAN, V2X, Satellite: Downlink/Uplink, UWB, RADAR system.
- Novel FSS_DWA_ of 5 × 5 repetitive structure with a wideband bandwidth of 1.742–11.78 GHz is placed below the MIMO_MBA_ antenna. The maximum peak-realized-gain enhances by 7.37dBi.
- The SAR_DWA_ analysis results are useful for the proposed MIMO_DWA_-FSS_DWA_ in the On-body application.
- The conformal characteristics of the MIMO_DWA_ antenna are useful in flexible electronics.

## Antenna configuration

### The optimal dimensions

**Fig. 1:**
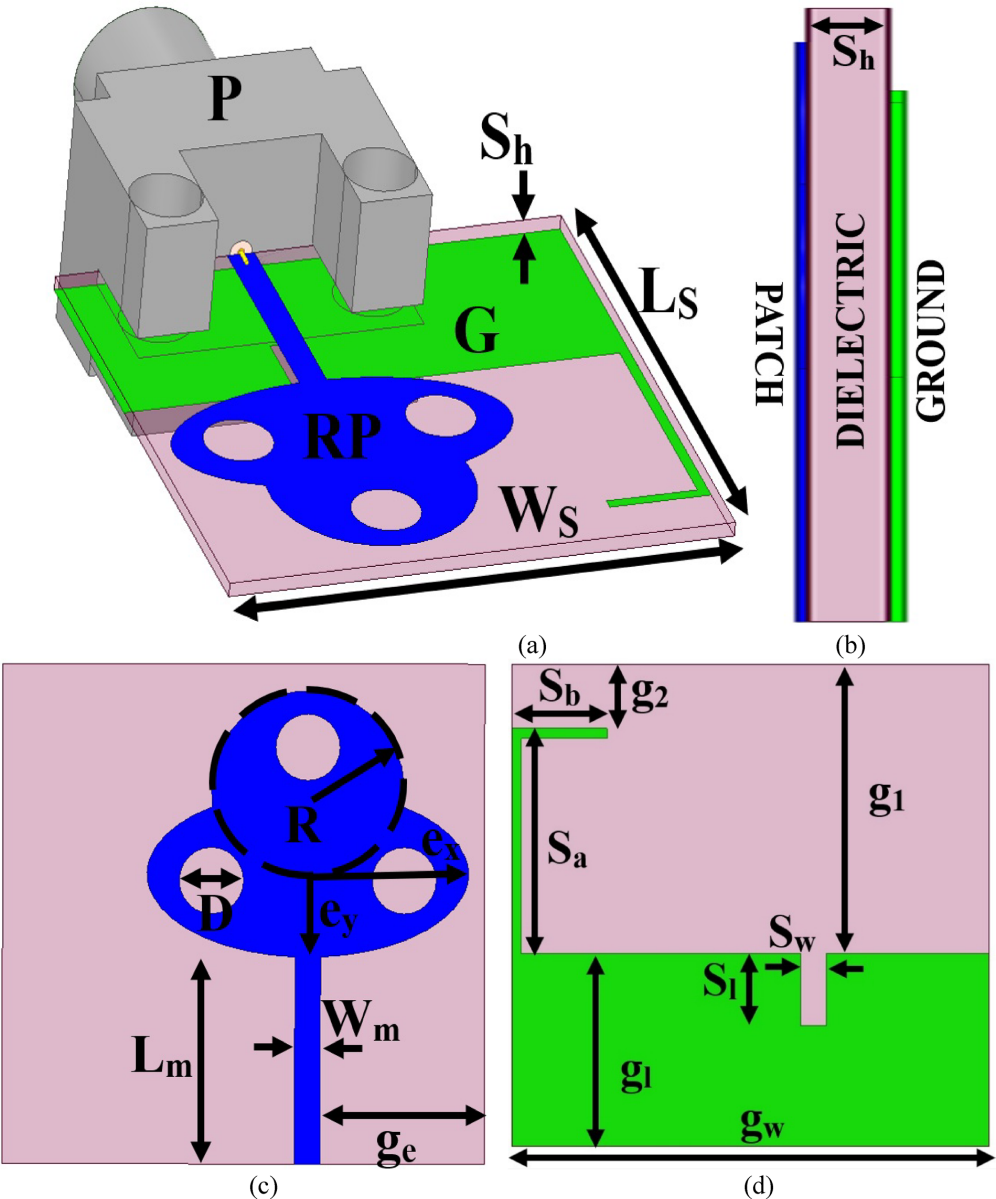
The proposed single-port antenna (**a**) Isometric details (**b**) Side view details (**c**) Front-view optimal dimensions (**d**) Ground view optimal dimensions

**Fig. 2:**
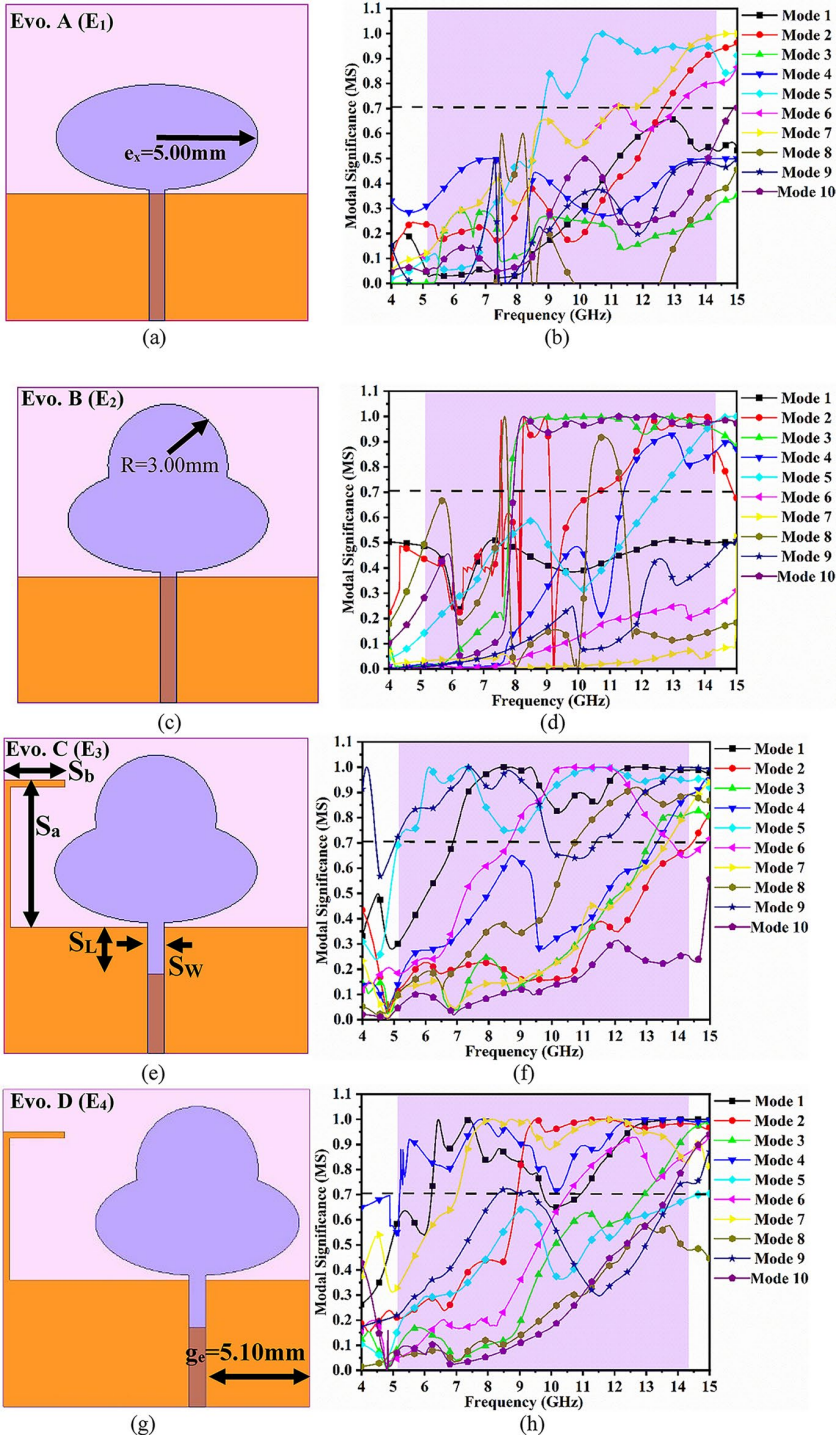

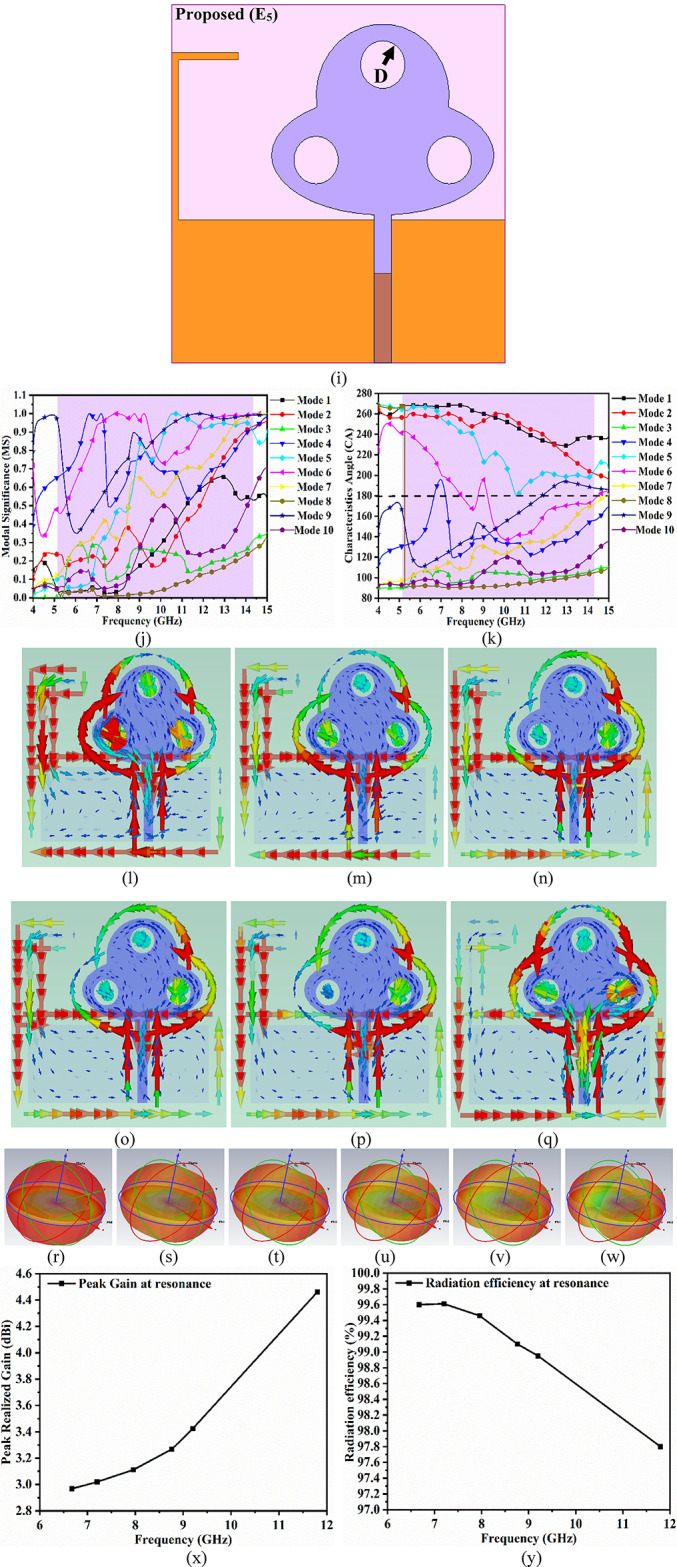
Evolution of the antenna model using characteristics-mode-analysis for 10-modes (**a**)-(**b**) Evo. A (E1) Malwith 10-mode modal-significance modes (**c**)-(**d**) Evo. B (E2) with 10-mode modal-significance modes (**e**)-(**f**) Evo. C (E3) with 10-mode modal-significance modes (**g**)-(**h**) Evo. D modes (**i**)-(**k**) Proposed antenna (E5) with 10-mode modal-significance and characteristics-angle; SCD of proposed antenna at (**l**)-(**q**) 6.67 GHz, 7.20 GHz, 7.958 GHz, 8.76 GHz, 9.20 GHz 11.80 GHz; 3-D radiation at (**r**)-(**w**) 6.67 GHz, 7.20 GHz, 7.958 GHz, 8.76 GHz, 9.20 GHz 11.80 GHz; PRG (dBi) and Radiation efficiency at (x)-(y) 6.67 GHz, 7.20 GHz, 7.958 GHz, 8.76 GHz, 9.20 GHz 11.80 GHz.

Figure1 illustrates the single-port ultra-wideband antenna for dual-wideband applications. Figure 1a shows the isometric details with Rogers-dielectric as the substrate used for designing the antenna component. The overall volume of the dielectric is W_s_×L_s_×S_h_ mm^3^ with a radiating patch on the top surface and ground on the opposite surface. The radiating patch, **RP**, is attached to a 50Ω microstrip transmission line, and the feed is connected to the SMK connector, **P**. Figure 2b shows the side-view of the antenna substrate with printed patch & ground with thickness of the substrate S_h_ mm and the copper thickness of 0.035 mm. Figure 1c corresponds to the front view with optimal dimensions. The patch is an ellipse of major/minor radius **e**_**x**_ mm and **e**_**y**_ mm with eccentricity ratio **e**_**x**_/**e**_**y**_=0.50 mm.

The elliptical patch is merged with a circular shape of radius R mm. The radiating patch, RP, is connected to a 50Ω-feed with a dimension of W_m_×L_m_ mm^2^. The radiating patch, RP, is etched by three circular slots of diameter D mm. The radiating patch with feedline is offset by the distance of g_e_ from the edge of the substrate. Figure 1(d) shows the ground printed on the opposite surface of the patch. The ground is rectangular-shaped with dimensions of g_w_×g_l_ mm^2^. The ground is etched by a rectangular slot of dimension S_w_×S_l_ mm^2,^ which is also offset placed beneath the microstrip-feedline. Additionally, the reflector is attached to the ground of dimension (S_a_+S_b_) mm. All the dimensions are optimized and are tabulated in Table 1.

**Table 1:** Optimal dimension values.

Par.	Value (mm)	Par.	Value (mm)	Par.	Value (mm)
W_s_	15.0	e_y_	2.50	L_m_	6.20
L_s_	15.0	R	3.00	g_1_	9.00
S_h_	0.254	S_a_	7.00	g_2_	2.00
S_l_	2.25	S_b_	3.00	g_e_	5.10
S_w_	0.80	D	2.00	g_w_	15.0
e_x_	5.00	W_m_	0.80	g_l_	6.00

### Evolution of antenna using characteristics model analysis (CMA) & the equivalent circuit model analysis (ECM)

Figure 2 shows the evolution of the single-port antenna configuration using characteristics-mode-analysis (CMA), where 10 modes are applied and the corresponding behavior is noted.

The single-port multi-band antenna in the FR1-band is subjected to mode-analysis known as Characteristics-Mode-Analysis (CMA_MBA_), where the key parameters, including modal-significance and characteristics-angle, are evaluated without giving any source signal as input. The Eigen-current is related to the impedance by the following Equations given below^48–50^.

1 $$\:Z=R+jX$$

2 $$\:R=\frac{Z+{Z}^{*}}{2}$$

3 $$\:X=\frac{Z-{Z}^{*}}{2}$$

The **Z** is the total net impedance offered by the antenna, with **R** corresponding to the real value of the impedance (without any loss), and $\:\pm\:$**jX** is the imaginary or lossy impedance, with **+jX** corresponding to the inductive nature and **-jX** capacitive nature. The higher the value of $\:\pm\:$**jX**, the more the antenna stores the energy instead of radiating electromagnetic waves. Hence, Eq. (1) needs to be more approximated to **Z**$\:\cong\:$**R**. On the other hand, Eqs. (2) and (3) corresponding to R and X indicate the average impedance value and differences of the real-imaginary values of the net impedance.

The term $\left\langle {E_{{\tan }}^{i} ~\left( r \right),~J_{n} } \right\rangle$ Corresponds to the model excited coefficient, and hence, the modal significance is derived as

4 $$\:MS=\left|\frac{1}{1+j{\lambda\:}_{n}}\right|$$

The modal significance (MS) is important as it relates to or ease of measuring the operational bandwidth or half-power bandwidth. $\:\left(\frac{1}{\sqrt{2}}=0.707\right)$ is given by

5 $$\:BW=\frac{{f}_{H}-{f}_{L}}{{f}_{res}}$$

When f_H_=f_L_ at resonance,

MS(f_o_(resonance) = 1

6 $$\:MS\left({f}_{H}={f}_{L}\right)=\left|\frac{1}{1+j{\lambda\:}_{n}}\right|=\frac{1}{\sqrt{2}}=0.707$$

Thus.

MS > 1/$\:\sqrt{2}$ corresponds to a significant mode and MS < 1/$\:\sqrt{2}$ signifies a non-significant mode.

The crossover angle at resonance corresponds to $\:{\varphi\:}_{n}=180^\circ\:$ and is mathematically calculated as

7 $$\:{\varphi\:}_{n}=180^\circ\:\:(1-\frac{1}{\pi\:}\:arc\:\left(tan{\lambda\:}_{n}\right))$$

The Eigenvalues (λ_n_) state that the storage of either magnetic or electric energy depends on the Eigenvalues with conditions λ_n_ > 0 for magnetic Energy, λ_n_ = 0 for the resonance condition, and λ_n_ < 0 for Electric Energy.

**Fig. 3:**
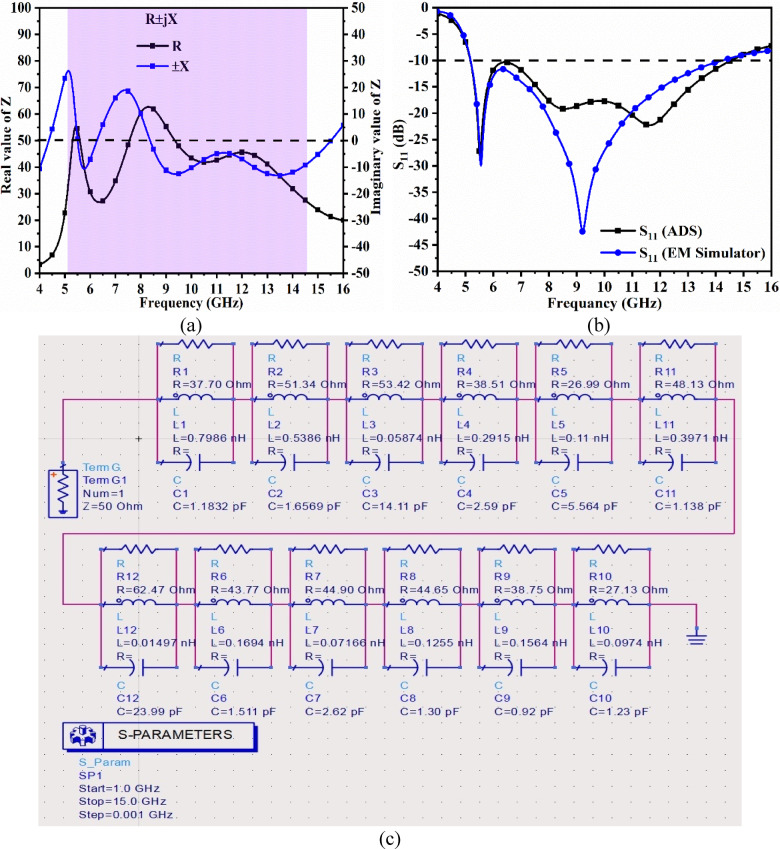

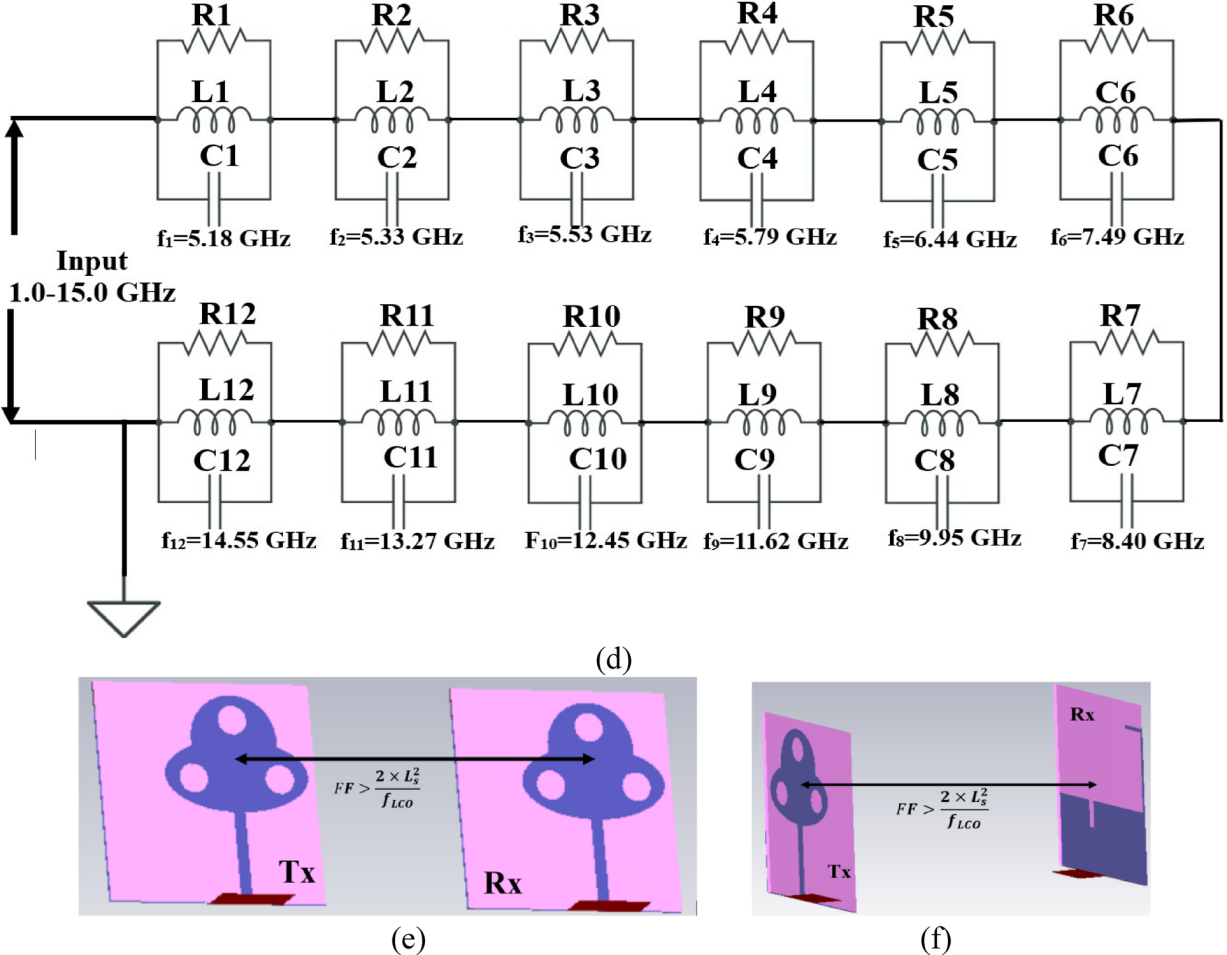

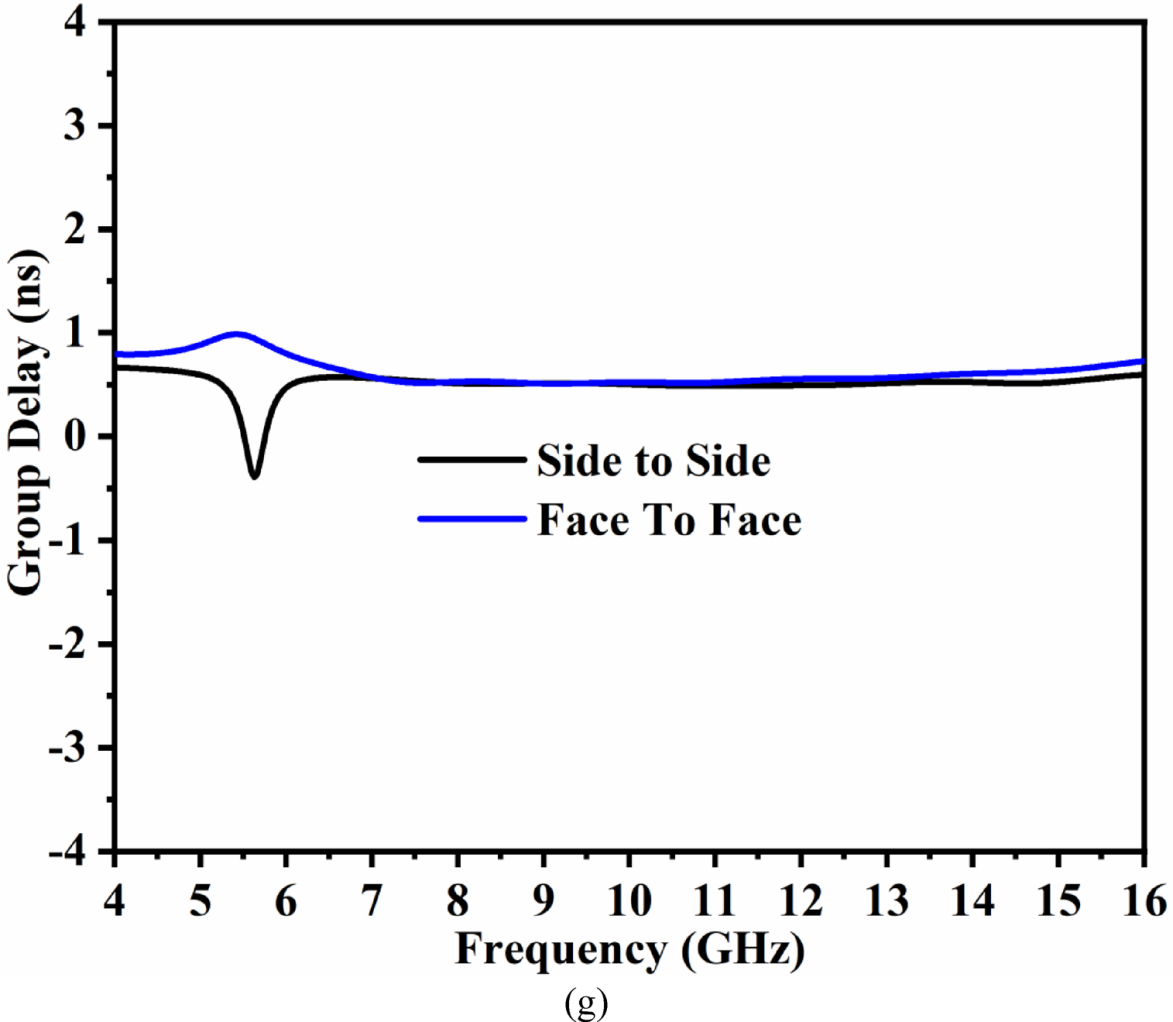
Equivalent Circuit Model (**a**) Impedance-plot (**b**) S_11_ comparison (**c**) Conceptual ADS Model (**d**) Circuit model; Group-delay analysis (**e**) Side-to-side arrangement (**f**) Face-to-Face (**g**) Group-delay.

The antenna shown in Fig. 1 is the single-port configuration achieving wider impedance bandwidth, which has been shown by simulated and equivalent circuit model S_11_ -10.0dB result in Fig. 3b. The final version of the antenna is achieved by an initial simple elliptical patch and ground, through iterations to achieve the required antenna. This is known as the evolution (Evo.) of the antenna shown in Fig. 2. Figure 2a corresponds to the printing of the elliptical patch on the top surface and partial ground on the opposite surface of Rogers dielectric. The patch is elliptical with a major radius, e_x_=5.00 mm, and this is Evo. A (E_1_) and is calculated from the following equations^51^.

8 $$\:{f}_{r}=\frac{15}{\pi\:e{e}_{xeff}}\sqrt{\frac{q}{{\epsilon\:}_{r}}}$$

9 $$\:{e}_{xeff}={e}_{x}{\left[1+\left(\frac{2{S}_{h}}{\pi\:{\epsilon\:}_{r}{e}_{x}}\right)p\right]}^{\frac{1}{2}}$$

10 $$\:p=ln\left(\frac{{e}_{x}}{2{S}_{h}}\right)+\left(1.41{\epsilon\:}_{r}+1.77\right)\frac{{S}_{h}}{{e}_{x}}\left(0.268{\epsilon\:}_{r}+1.65\right)$$

11 $$\:q=-0.0049e+3.7888{e}^{2}-0.7278{e}^{3}+2.31{e}^{4}$$

Where f_r_ is the resonance frequency (GHz), e_x_ is the major radius of the elliptical patch, and S_h_ is the width of the dielectric.

Figure 2b shows the CMA simulated result of antenna E_1,_ where the antenna generates a bandwidth of 9.05–20.0 GHz with Mode 2, Mode 5, Mode 6, and Mode 7 being the most significant. However, the objective is to achieve a wideband antenna that can find applications for WLAN, X-band satellite, and UWB.

Hence, the need arises for modification, which is Evo. B (E_2_) antenna achieved by merging a circular patch of radius *R* = 3.00 mm, which is shown in Fig. 2c. This modification is applied to the radiating patch with better impedance matching, achieving a bandwidth of 7.38–19.04 GHz with the most significant modes corresponding to Mode 2, Mode 4, Mode 5, Mode 8, and Mode 10 noted from Fig. 2d. The next evolution, Evo. C shown in Fig. 2e is applied on the ground with the etching of a rectangular slot of dimension S_w_×S_l_=0.80 × 2.25 mm^2^ and the addition of a stub of length (S_a_+S_b_) = 10.0 mm. These two modifications observe much better matching of the impedance with the in-depth resonance of 7.92 GHz (S_11_=-14.15 dB) and 12.94 GHz (S_11_=-27.71 dB), and the contributing most significant modes are Mode 1, Mode 2, Mode 3, Mode 4, Mode 5, Mode 6, Mode 7, Mode 8, and Mode 9. The next stage of transformation is Evo. D (E_4_) with an offset of the patch and an etched rectangular slot in the ground as shown in Fig. 2g. This further achieves a matched − 10.0 dB impedance bandwidth of 5.19–14.02 GHz with an offset of g_e_=5.10 mm from the edge of the dielectric. The most dominant modes in this evolution are Mode 1, Mode 2, Mode 3, Mode 4, Mode 6, and Mode 7 as shown in Fig. 2h. The final version of the antenna shown in Fig. 2i, where etching of three circular slots on a patch with a diameter of D = 2.00 mm, which not only improves the matching of impedance but also achieves an operational − 10.0dB bandwidth of 5.18–14.18 GHz, including applications for WLAN band, Ultra-wideband, X-band and RADAR applications in Ku-band. The achieved bandwidth is supported by CMA, including the calculation of modal significance shown in Fig. 2j and characteristic angle shown in Fig. 2(k). The five most dominant modes corresponding to Mode 2, Mode 4, Mode 5, Mode 6, Mode 7 and Mode 9 generates resonance with impedance of (50 + j0)Ω at 6.67 GHz, 7.20 GHz, 7.958 GHz, 8.76 GHz, 9.20 GHz and 11.80 GHz which is noted from characteristics angle graph shown in Fig. 2k.

The surface-current-density (SCD) distribution and the corresponding 3D-radiation patterns are also plotted for the six resonance values, which coincide with the 180^◦^ line of the characteristics angle graph shown in Fig. 2k. For all six resonance values of frequency corresponding to 6.67 GHz, 7.20 GHz, 7.958 GHz, 8.76 GHz, 9.20 GHz, and 11.80 GHz, the SCD shows an even distribution at the center of the patch, achieving high efficiency. Also, the antenna maintains desirable omnidirectional and dipole patterns in H- and E-plane for all the aforementioned six frequency values. Figure 2x and Fig. 2y also show the graph that calculates the peak-gain and radiation efficiency at six resonances and are tabulated in Table 2.

**Table 2:** Peak-gain and radiation efficiency at six-resonance values.

Frequency (GHz)	Peak gain (dBi)	Radiation efficiency (%)
6.67	2.97	99.60
7.20	3.10	99.61
7.958	3.11	99.46
8.76	3.27	99.10
9.20	3.42	98.95
11.80	4.46	97.80

Table 2 records the value of peak-gain and radiation efficiency at six-resonance values, with peak-gain ranging between 2.97dBi-4.46dBi. Also, the radiation efficiency of the antenna is more than 97.80% which is due to the resonance condition, and also the antenna does not store any electric or magnetic energy (X_l_=X_c_=0).

Figure 2 illustrates the analysis of the single-port antenna shown in Fig. 1. The antenna achieves a wide impedance bandwidth inclusive of multiple resonances within the operational bandwidth. Each resonance can be treated as a parallel-connected RLC lumped circuit connected in series, which is represented by the general wideband Eq. (12)^48^, where n represents an infinite number of resonance frequencies or frequency values within the operating bandwidth and corresponds to a series-connected parallel RLC circuit. Thus, the impedance needs to be calculated where the lumped-RLC components are calculated from Eq. (13)^48^to Eq. (15)^48^. The net impedance represented by Z = Z_1_ + Z_2_ + Z_3_ + Z_4_+Z_5_ + Z_6_ + Z_7_+Z_8_+Z_9_+Z_10_+Z_11_+Z_12_=Z_L_ is plotted in Fig. 3(a), where in the − 10.0dB bandwidth, the Re-impedance grazes the 50Ω impedance and $\:\pm\:$**jX** corresponds to 0Ω imaginary impedance. The twelve values of frequency values are chosen from the S_11_-graph shown in Fig. 3b to realize the equivalent circuit model. Each frequency value will correspond to parallel-connected lumped-element RLC components evaluated from Eq. (27) to Eq. (29). The frequency values are tabulated in Table 3 with corresponding S_11_ dB, R Ω, and $\:\pm\:$**jX** Ω needed to calculate the lumped RLC components shown in Fig. 3d. The conceptual model with values of L and C is tabulated in Table 3, which is obtained from Eqs. (28) and (29). The equivalent circuit model is drawn in an ADS RF simulator with all the calculated values of lumped components. The simulated Saa obtained is plotted in Fig. 3b and is compared with S_11_ obtained from the EM-simulator. This analysis gives more insight into understanding the impedance matching of the wideband antenna.

**Table 3:** Lumped Component Values (RLC).

Frequency (GHz)	S_11_ (dB)	Img.	*R* (Ω)		L (nH)		C (pF)	
5.18	-10.05	25.98	R1	37.70	L1	0.7986	C1	1.1832
5.33	-15.10	18.03	R2	51.34	L2	0.5386	C2	1.6569
5.53	-28.30	2.04	R3	53.42	L3	0.05874	C3	14.11
5.79	-15.12	10.60	R4	38.51	L4	0.2915	C4	2.59
6.44	-10.35	4.45	R5	26.99	L5	0.11	C5	5.564
7.49	-14.52	18.68	R6	48.13	L6	0.3971	C6	1.138
8.40	-19.09	0.79	R7	62.47	L7	0.01497	C7	23.99
9.95	-17.71	10.59	R8	43.77	L8	0.1694	C8	1.511
11.62	-22.29	5.23	R9	44.90	L9	0.01766	C9	2.62
12.45	-18.59	9.82	R10	44.65	L10	0.1255	C10	1.30
13.27	-14.33	13.04	R11	38.75	L11	0.1564	C11	0.92
14.55	-10.00	8.90	R12	27.13	L12	0.0974	C12	1.23

12 $$\:{Z}_{L}=\sum\:_{j=1}^{n}\frac{j\omega\:{R}_{j}{L}_{j}}{{R}_{j}\left(1-{\omega\:}^{2}{L}_{j}{C}_{j}\right)+j\omega\:{L}_{j}}$$

13 $$\:f=\frac{1}{2\pi\:\sqrt{LC}}$$

14 $$\:L=\frac{imag\left({Z}_{11}\right)}{2\pi\:{f}_{o}}$$

15 $$\:C=\frac{1}{{\left(2\pi\:{f}_{o}\right)}^{2}L}$$

Group delay, which is another time-domain response, is defined as the negative derivative of phase change irrespective of frequency. It is also known that the wave incident on the antenna will include multiple frequency components (Fig. 3e-g), and group delay signifies the dispersive nature of the device. The group delay also validates the linear response of the phase in the complete far-field region. The variation of the group delay should not be more than $\:\pm\:$1ns, which indicates a linear phase for all the frequency ranges. The group delay is calculated from the following Eq. 

16 $$\:{\tau\:}_{g}\left(\omega\:\right)=-\frac{d\varphi\:\left(\omega\:\right)}{d\omega\:}=\frac{d\varphi\:\left(\omega\:\right)}{2\pi\:df}$$

Where φ – transmitted phase response

ω – frequency in radians.

Figure 3e and Fig. 3f show the arrangement of transmitter and receiver (identical proposed antenna) placed at a far-field (FF) distance of 100.0 mm, with the arrangement corresponding to side-to-side and face-to-face. The group delay is calculated from Eq. (16) with the variation below $\:\pm\:$0.5ns and ensures linear phase-response.

### Parametric study

**Fig. 4:**
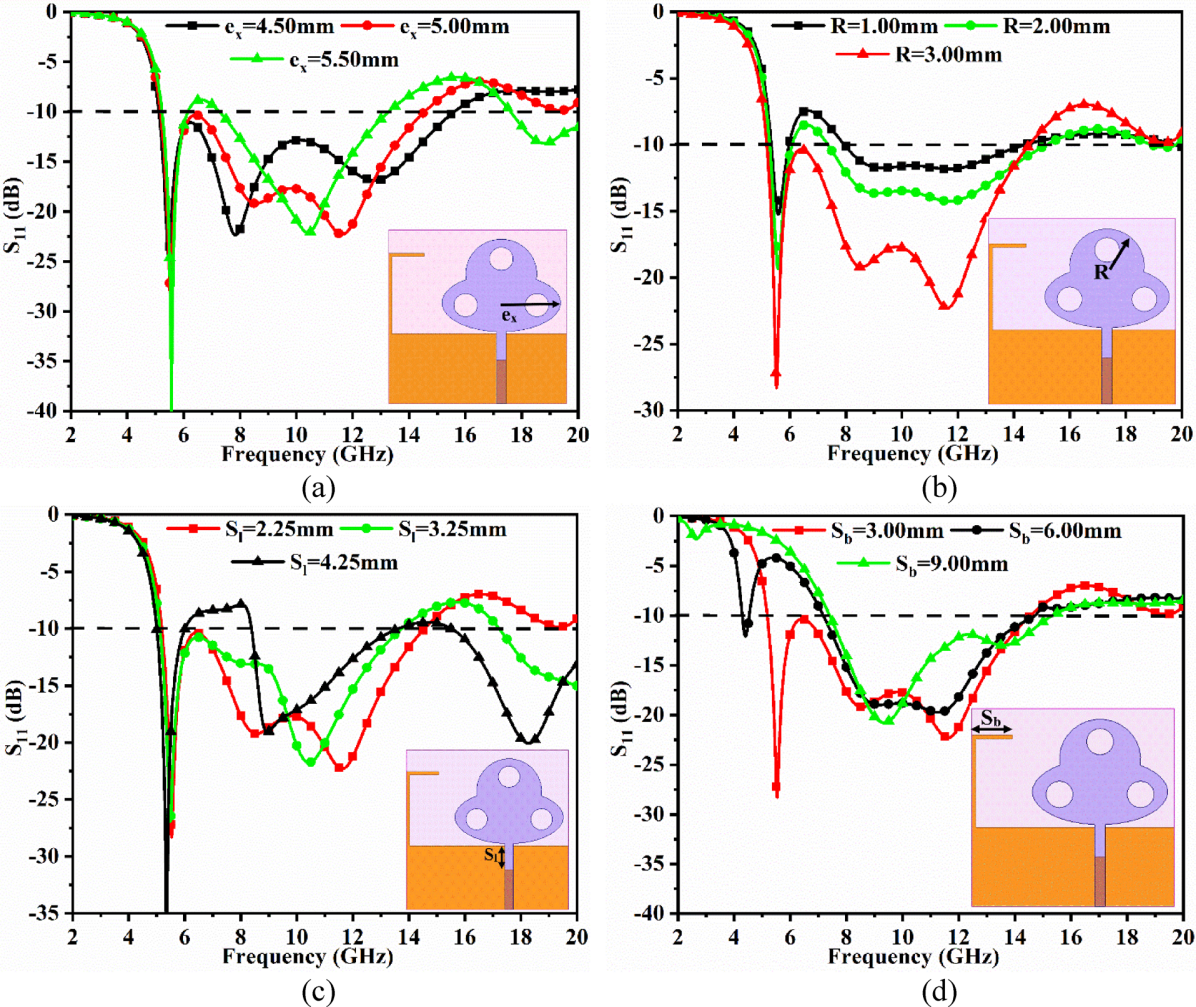
Parametric analysis (**a**) e_x_ (**b**) R (**c**) S_l_ (**d**) S_b_.

The parameter analysis of the optimized dimensions tabulated in Table 1 is achieved by varying the values of key parameters. The four key parameters include e_x_ (major radius of the elliptical patch), **R** (radius of the merged circular patch with radius), S_l_ (etched ground slot length), and Sb (length of the attached stub to ground).

The first parameter, e_x_ is varied for three values corresponding to 4.50 mm, 5.00 mm, and 5.50 mm, respectively. The value of e_x_=4.50 mm observes poor matching between 10.0 and 16.0 GHz as noted in Fig. 4a, indicating the reduction of the size of the elliptical patch, which also deteriorates the impedance matching. On the other hand, for a large size of the elliptical patch, e_x_=5.50 mm, the − 10.0dB bandwidth reduces to two bands, one narrow and the other wide-band. However, all the required applications are not included, and hence, for e_x_=5.00 mm, the wider impedance bandwidth is achieved.

The second parameter, the radius of the circular patch, **R**, also impacts the matching of the working impedance-bandwidth. The value of *R* = 1.00 mm and *R* = 2.00 mm increases the size of the circular patch, thereby increasing the area for the flow of current, but it is not enough to achieve a good impedance match. In both cases (*R* = 1.00 mm and 2.00 mm), the matching of impedance is very poor as observed in Fig. 4b for bandwidth ranging between 7.00 and 15.0 GHz. For a radius of circular-patch corresponding to *R* = 3.00 mm, with maximum optimized area, achieves − 10.0dB bandwidth of 5.18–14.55 GHz.

The two-parameter, e_x_, and R correspond to the radiating patch, while the other two parameters, S_l_ and S_b_, correspond to the ground parameter printed on the opposite surface of the patch. The parameter, S_l_, which is the length of the etched slot in the ground, is changed from 2.25 mm to 4.25 mm with a step size of 1.00 mm. As per the observations from Fig. 4c, the value of S_l_=3.25 mm loses its matching of impedance matching between 7.00 and 9.00 GHz in comparison to S_l_=2.25 mm, which is the optimal dimension. The value of S_l_=4.25 mm, the partial ultra-wideband is filtered due to deterioration of the matching of impedance matching, and hence, the application band in X-band is eliminated.

Finally, the stub length, S_b_ mm, which is added to act as a reflector, is varied between 3.00 mm and 9.00 mm with a step size of 3.00 mm between the values. The values of Sb = 6.00 mm and 9.00 mm, have an impact on the lower-cut-off frequency, which reduces in both cases as observed in Fig. 4d. However, for a value of S_b_=3.00 mm, the required operational bandwidth of 5.18–14.55 GHz is achieved.

## MIMO antenna configuration

The MIMO technology has numerous advantages over the deployment of a single-port antenna system in the fading channel, which include.

i. Increase in data rates: The enabling of the multiplexing offers different data to be transmitted at the same time over the fading channel.
ii. The improvement in the spectral efficiency is increased, which allows the transmission of more bits per unit of the operating bandwidth.
iii. Also, MIMO_DWA_ technology deploys spatial diversity in the proposed work, which not only mitigates fading but also the inter-element radiation interference.
iv. The utilization of MIMO_DWA_-Diversity gain reduces the effects of multi-path fading of the signals.

Also, the MIMO_DWA_ technology, based on the bandwidth achieved for the proposed work, finds applications in.

- X-band satellite communication.
- IoT and Smart Cities in the UWB range.
- Autonomous vehicles and V2X communication in dedicated short-range-communication (DSRC) with frequency bandwidth of 5.850–5.925 GHz (5.90 GHz).

On the other hand, the printing of a greater number of radiating patches has the advantage of increased bandwidth and efficiency, which is given by the Shannon-Hartley channel-capacity theorem given by^48^

17 $$\:{CC}_{\text{D}\text{W}\text{A}}={M}_{WDB}\times\:\varDelta\:BW\times\:\left(1+\frac{S}{N}\right)$$

**Fig. 5:**
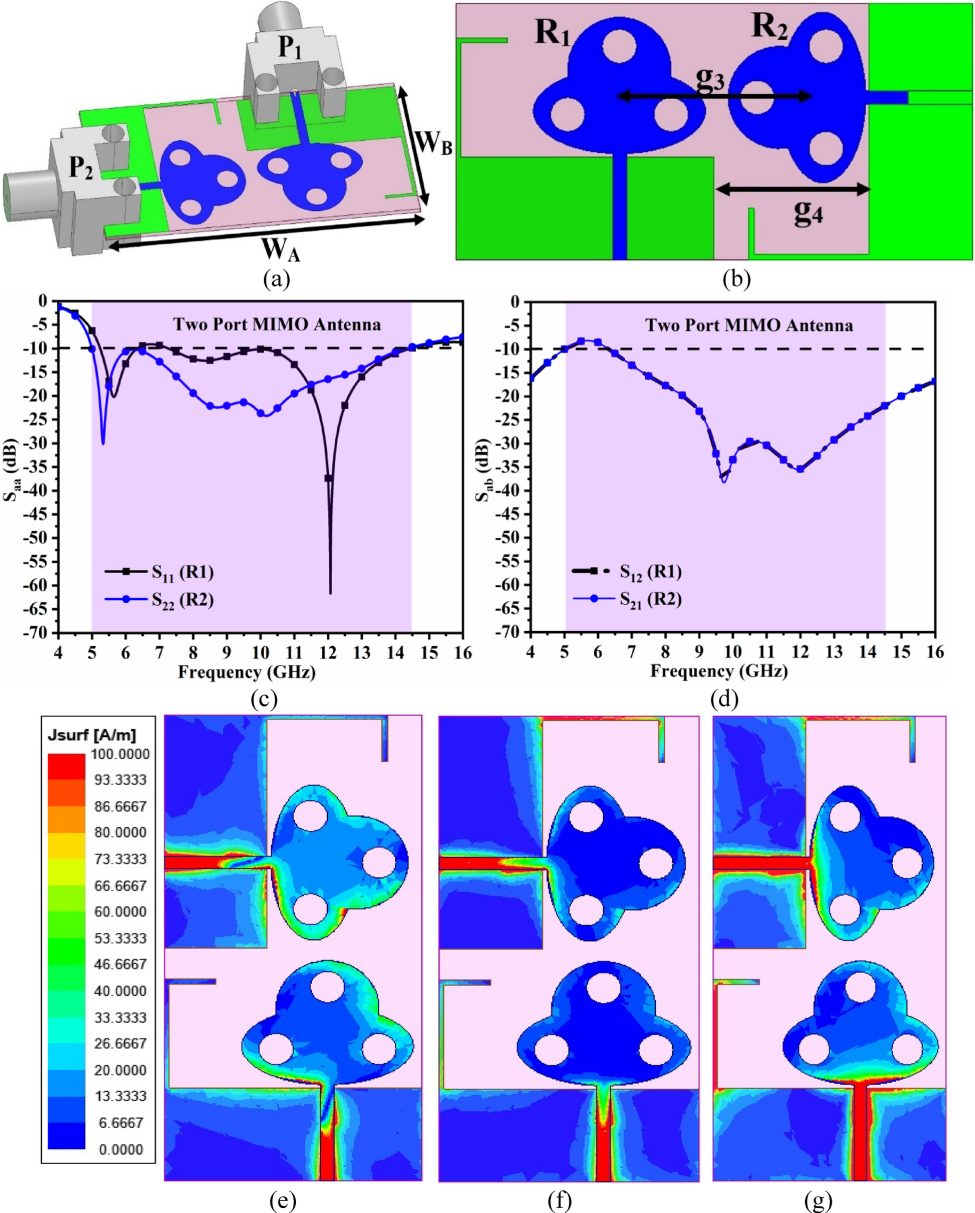
Two-port MIMO_MBA_ antenna (**a**) Isometric-view (**b**) Optimal-dimension (patch-ground); S-parameter (**c**) Reflection-coefficients (**d**) Transmission-coefficients SFD at (e) 5.50 GHz (f) 7.50 GHz (g) 12.0 GHz.

Where M_DWA_ – no. of radiating elements.

ΔBW – operational bandwidth.

S/N – signal-to-noise ratio of the channel.

Equation (17)^48^ signifies that the bandwidth of the system will increase due to an increase in radiating elements (M_MBA_), and the SNR should be high by suppressing noise as low as possible.

Figure 5 illustrates the development of the proposed two-port MIMO_DWA_ antenna for more accuracy and less loss of data transmission over the fading channel. The single-port antenna discussed in Sect. 2 showed the capability of achieving multi-band applications with a bandwidth ranging between 5.18 GHz and 14.55 GHz. However, by increasing the number of identical radiating paths, the speed of data transfer is increased, which motivates for implementation of multi-input-multi-output_DWA_ (MIMO_DWA_) technology. Figure 5a shows the 3-D view of the two-port MIMO_DWA_ antenna with the total dimension of the antenna corresponding to W_A_×W_B_=30 × 15 mm^2^. The two individual antennae with the respective ground are placed orthogonally concerning each other and are connected to input ports P_1_ and P_2,_ respectively. Figure 5b shows the optimal dimension with distances between the patch and the ground. The inter-spacing between the two radiating patches, R_1_ and R_2_ is maintained for better isolation. Also, the spacing of g_4_ = 9.00 mm is observed between the two grounds of the respective two-element radiating patch. Figure 5c and Fig. 5d record the performance of reflection and transmission coefficients. The proposed two-port configuration maintains the S_11_ bandwidth for the R_1_ antenna of 5.24–14.38 GHz, and for R_2_, the S_22_ bandwidth is 5.00–14.30 GHz as noted in Fig. 5c. Figure 5d records the isolation between the two radiating antennae, R_1_ and R_2,_ which is more than 10.0dB in the entire operating bandwidth and is better between the bandwidth of 7.00–14.38 GHz, which is more than 15.0dB. The effect of mutual coupling or the amount of interaction of the radiating EM-wave by each radiating patch is analyzed by studying the surface-current density of the individual radiating antenna at key application frequency values. Figure 5e, Fig. 5f, and Fig. 5g show the simulated SFD_DWA_ for frequency values of 5.50 GHz, 7.50 GHz, and 12.0 GHz with the condition that both the input port is excited simultaneously with the power of 1 W. In all the simulated three frequency values, the maximum SFD_DWA_ is observed to be concentrated within the transmission line, which indicates that good matching of impedance is achieved between the input port and the radiating patch, as the transmission line serves as a signal carrier and not part of any radiation. However, all the signals received by the antenna at 5.50 GHz, 7.50 GHz, and 12.0 GHz are radiated by each of the radiating patches, R_1_ and R_2,_ respectively.

**Fig. 6:**
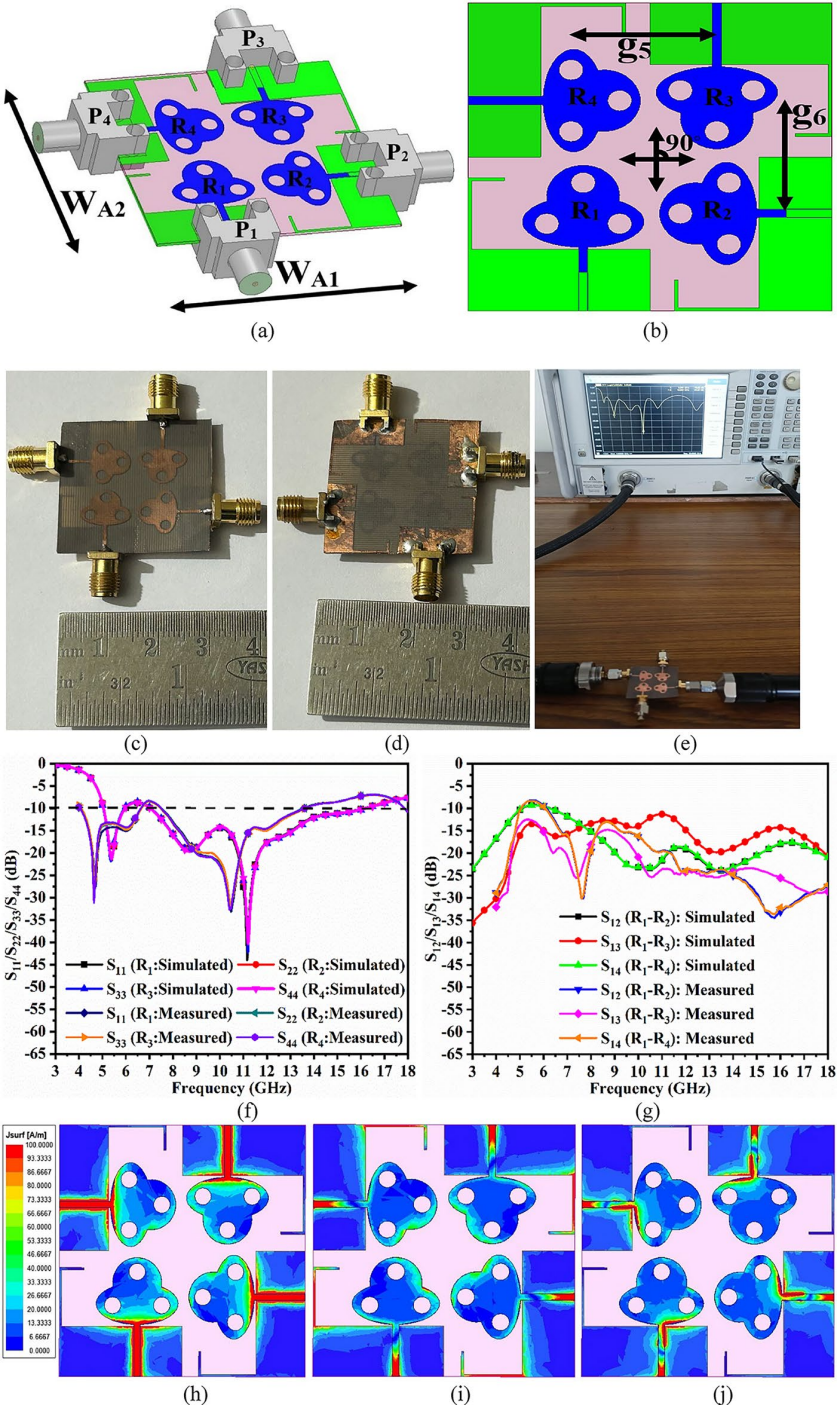

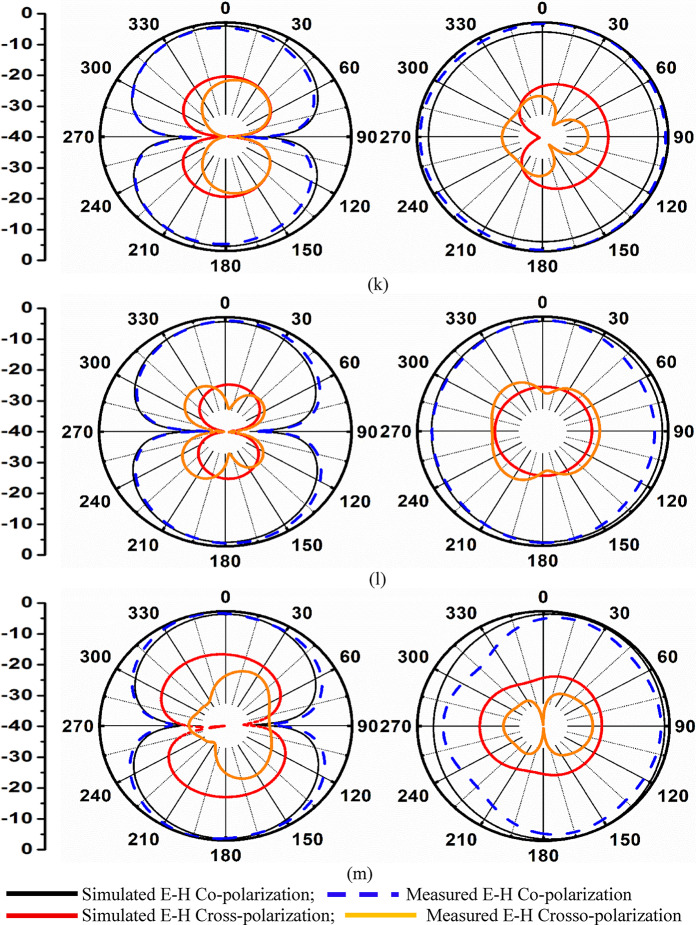
Four-port MIMO_MBA_ antenna (**a**) Isometric-view (**b**) Optimal-dimension (patch-ground) (**c**)-(**d**) Fabricated photograph of Patch-ground (**e**) VNA Connected to prototype (**f**) Simulated-Measured reflection-coefficients (**g**) Simulated-Measured transmission-coefficients; SFD at (**h**) 5.50 GHz (**i**) 7.50 GHz (**j**) 12.0 GHz; Simulated-Measured 2D-Radiation in E-H plane at (**k**) 5.50 GHz (**l**) 7.50 GHz (**m**) 10.0 GHz.

The merit of increasing the number of radiating elements has several advantages over the single-port configuration, which has been discussed in the two-port configuration. Also, a faster data rate is achieved by adding an identical radiating patch and forming a four-port MIMO_DWA_ configuration as shown in Fig. 6. The four-port MIMO_DWA_ configuration is the extended version of the two-port MIMO_DWA_ antenna, with all the radiating patches marked as R_1_, R_2_, R_3,_ and R_4_ placed in orthogonal sequence as shown in Fig. 6a. Each of the antenna elements is connected by SMK-port identified as P_1_, P_2_, P_3,_ and P_4,_ respectively, with a dimension of the antenna, W_A1_×W_A2_=30 × 30 mm^2^. Figure 6b illustrates the front view of the antenna with an inter-spacing between the adjacent pair of g_5_ = g_6_ = 11.50 mm. Figure 6c and Fig. 6d show the photograph of the fabricated four-port MIMO antenna with front and ground views. More accuracy in maintaining the exact dimensions is achieved by using the photolithographic method of fabrication. Also, Fig. 6(e) includes a photograph of the measurement of the prototype connected to the VNA with an S-parameter measurement display. The comparison of S-parameters with the plot of simulated and measured reflection & transmission coefficients is also plotted in Fig. 6.

**Table 4:** Simulated and measured result comparison of Four-Port MIMO without FSS.

4-Port MIMO	Simulated		Measured	
Bandwidth (GHz)	Band A	Band B	Band A	Band B
	5.05–5.98	7.03–16.12	4.04–6.64	7.44–16.60
Isolation (dB)	Band A	Band B	Band A	Band B
	> 15.0	> 10.0	> 12.0	> 15.0

Figure 6f corresponds to a -10.0dB bandwidth obtained with the plot of all four ports corresponding to the graph of S_11_/S_22_/S_33_/S_44_. It can be observed that around 1 GHz of the input signal, ranging between 6.00 GHz and 7.00 GHz, is filtered, and the MIMO_MBA_ antenna produces almost overlapping bandwidths of 5.05–5.98 GHz, marked as Band-A, and 7.03–16.12 GHz, marked as Band-B. For the measured results shown in Fig. 6f, the fabricated MIMO antenna also produces two operating bands, Band-A with a bandwidth of 4.04–6.64 GHz and Band-B bandwidth of 7.44–16.60 GHz, respectively. The amount of interference between the four-radiating elements is also plotted in Fig. 6g. As per the observations from Fig. 6g, the simulated transmission coefficients observe isolation of more than 9.99dB between port-1-port2 and port1-port4. The isolation between ports 1 and 3 is much better in Band-A, with more than 15.0 dB. For Band-B, the simulated isolation is more than 10.0dB between port1—port3 and greater than 12.0dB for the remaining pair of ports. Figure 6g corresponds to measured isolation, which is more than 7.68dB for any pair of two-ports in Band-A and more than 15.0dB in Band-B. Table 4 records the comparison of the simulation and fabricated MIMO antenna, irrespective of operational bandwidths and the maximum isolation offered between the inter-spaced elements. Figure 6h, Fig. 6i and Fig. 6j include surface-current-density plots. It can be observed that for all three frequency values corresponding to 5.50 GHz, 7.50 GHz & 12.0 GHz, surface-current density is concentrated within the microstrip-transmission line, which carries the signal from the input port to the radiating patch. Also, Fig. 6h, Fig. 6i, and Fig. 6j conclude that there is very little interference among the neighboring radiating elements and justify the arrangement of the orthogonal sequence.

**Fig. 7:**
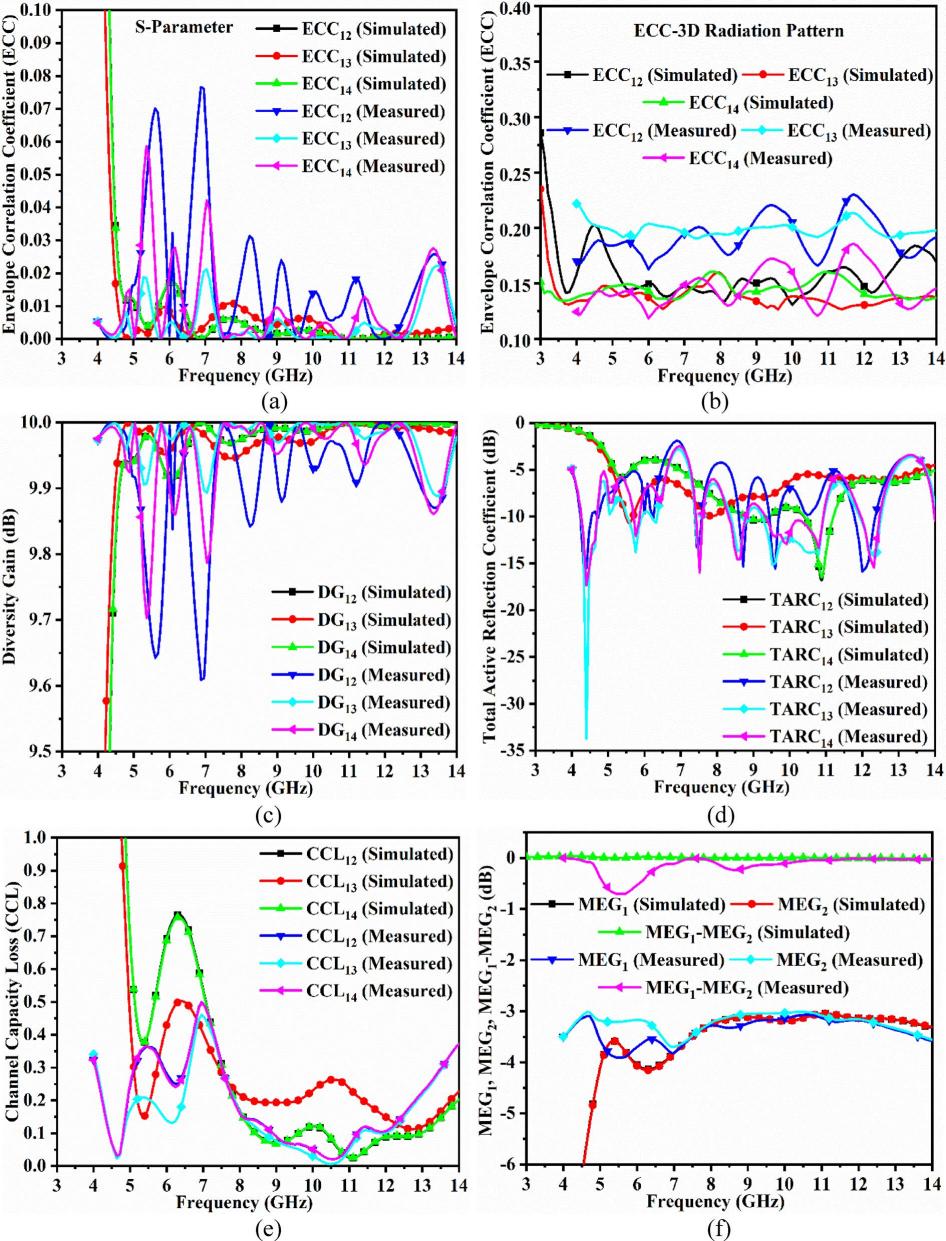

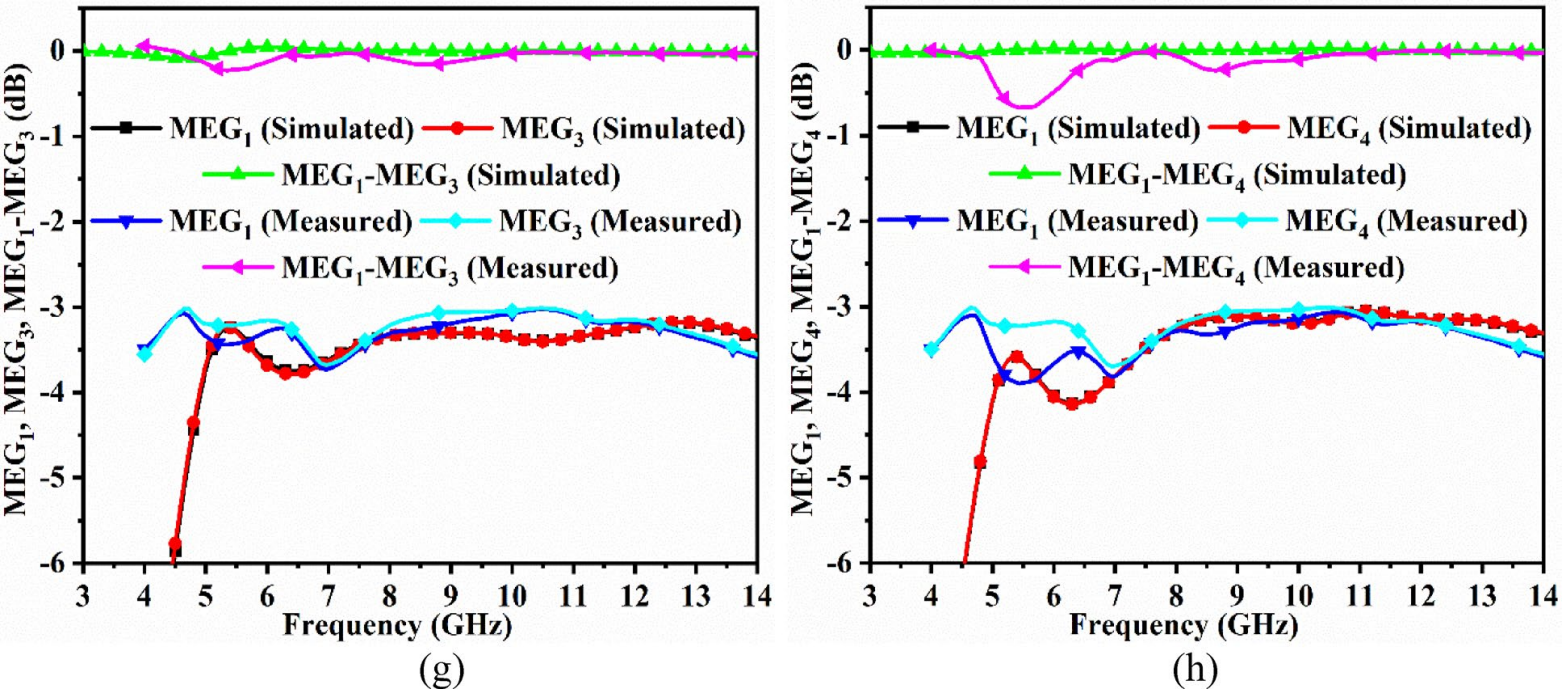
Four-port MIMO antenna diversity performance (**a**) Simulated-Measured envelope-correlation-coefficient of dual-band antenna (S-parameter method) (**b**) Simulated-Measured envelope-correlation-coefficient of dual-band antenna (3D Radiation method) (**c**) Simulated-Measured diversity-gain of dual-band antenna (**d**) Simulated-Measured total-active-reflection-coefficient of dual-band antenna (**e**) Simulated-Measured channel capacity loss of dual-band antenna (**f**) Simulated-Measured mean-effective-gain between port1-port2 of dual-band antenna (**g**) Simulated-Measured mean-effective-gain between port1-port3 of dual-band antenna (**h**) Simulated-Measured mean-effective-gain between port1-port4 of dual-band antenna.

The monopole-single feed antenna exhibited the capability of generating a wideband with not only compact but also dual-wideband antenna (DWA) applications. However, the importance of multiple numbers of radiating elements within the same antenna system, eliminating the fading problem, has been discussed and supported by the Shannon-Hartley capacity theorem, where the signal-to-noise ratio is another parameter of concern in achieving the reduced multi-path signals. However, this work focuses on the objective of a multi-four-port antenna configuration with a symmetrical structure, including S_DBA_ (S-parameter for dual-wideband antenna).

The reflection-coefficients from the above square S-matrix corresponds to S11_DWA_, S22_DWA_, S33_DWA_ and S44_DWA_ with remaining twelve S-parameters corresponding to S12_DWA_, S13_DWA_, S14_DWA_, S21_DWA_, S23_DWA_, S24_DWA_, S31_DWA_, S32_DWA_, S34_DWA_, S41_DWA_, S42_DWA_ and S43_DWA_ respectively corresponding to transmission coefficients. Each of the four-port S_11(MBA)_, S_22(DWA)_, S_33DWA),_ and S_44(DWA)_ corresponds to the S-parameter and − 10.0dB lower and upper cut-off frequency corresponding to the respective individual antenna element bandwidth. Also, the input signal fed to each of the radiating paths marked as R_1_, R_2_, R_3_, and R_4_ (Fig. 6a) radiates the signal, forming radiation patterns in E-H planes. However, due to the orthogonal orientation, the MIMO_DWA_ antenna achieves spatial diversity, and the diversity characteristics such as ECC_DWA_, DG_DWA_, TARC_DWA_, CCL_DWA,_ and MEG_DWA_ need to be evaluated for both simulated and measured S-parameters. The calculated diversity parameter within the − 10.0dB operational bandwidth needs to be within the specified standard values so that the proposed MIMO_MBA_ antenna can be deployed in the fading wireless channel for real-time applications.

**Table 5:** Diversity parameters comparison.

4-Port MIMO	Simulated		Measured		Threshold value
	Band A	Band B	Band A	Band B	
ECC	< 0.01	< 0.012	< 0.07	< 0.028	$\:\le\:$0.50
DG (dB)	> 9.958	> 9.982	> 9.996	$\:\cong\:$10.0	> 9.95
TARC (dB)	<-4.0	<-4.18	<-6.22	<-5.14	< 0.0
CCL (b/s/Hz)	< 0.38	< 0.25	< 0.36	< 0.14	< 0.40
MEG (dB)	$\:\cong\:$-3.0	$\:\cong\:$-3.0	$\:\cong\:$-3.0	$\:\cong\:$-3.0	$\:\cong\:$-3.0

The parameter, Envelope-Correlation-Coefficient_MBA_ (ECC_DWA_), signifies the amount of radiation interference between the neighboring antenna elements. In general, the value of ECC_MBA_ ranges between 0 and 1, with 0 indicating no interference and 1 corresponding to maximum interference. The ECC_DWA_ is evaluated either by the 3-D radiation-pattern method, with radiation in the E-plane considered between the two radiating elements represented by Eq. (18)^48^

18 $$\:{\gamma\:}_{c}=\frac{\underset{0}{\overset{2\pi\:}{\int\:}}\underset{0}{\overset{\pi\:}{\int\:}}\left(({XPRE}_{\theta\:.m}\left(\theta\:,\varphi\:\right)\:{E}_{\theta\:,s}^{*}\:\left(\theta\:,\varphi\:\right){P}_{\theta\:}\left(\theta\:,\varphi\:\right)+\:{E}_{\varphi\:.m}\left(\theta\:,\varphi\:\right)\:{E}_{\varphi\:,s}^{*}\:\left(\theta\:,\varphi\:\right){P}_{\varphi\:}\left(\theta\:,\varphi\:\right)\right)sin\theta\:\:d\theta\:\:d\varphi\:\:}{\sqrt{{\delta\:}_{m}^{2}}\sqrt{{\delta\:}_{s}^{2}}\:}$$

Here, $\:{\delta\:}_{m}^{2}$ sand $\:{\delta\:}_{s}^{2}$ Is the variance related to ports, which can be further mathematically expressed as^48^

19 $$\:\underset{0}{\overset{2\pi\:}{\int\:}}\underset{0}{\overset{\pi\:}{\int\:}}\left(({XPRG}_{\theta\:.m}\left(\theta\:,\varphi\:\right)\:{P}_{\theta\:}\left(\theta\:,\varphi\:\right)+\:{G}_{\varphi\:.m}\left(\theta\:,\varphi\:\right){P}_{\varphi\:}\left(\theta\:,\varphi\:\right)\right)\:$$

20 $$\:{G}_{\theta\:m}\left(\theta\:,\varphi\:\right)={E}_{\theta\:m\:}\left(\theta\:,\varphi\:\right){E}_{\theta\:s}^{*}(\theta\:,\varphi\:)$$

21 $$\:{G}_{\varphi\:m}\left(\theta\:,\varphi\:\right)={E}_{\varphi\:m\:}\left(\theta\:,\varphi\:\right){E}_{\varphi\:s}^{*}(\theta\:,\varphi\:)$$

22 $$\:{G}_{\theta\:s}\left(\theta\:,\varphi\:\right)={E}_{\theta\:s\:}\left(\theta\:,\varphi\:\right){E}_{\theta\:m}^{*}(\theta\:,\varphi\:)$$

23 $$\:{G}_{\varphi\:s}\left(\theta\:,\varphi\:\right)={E}_{\varphi\:s\:}\left(\theta\:,\varphi\:\right){E}_{\varphi\:m}^{*}(\theta\:,\varphi\:)$$

The complex electric field in the E-plane concerning θ and ϕ corresponds to $\:{E}_{\theta\:m}$, $\:{E}_{\varphi\:m}$ $\:{E}_{\theta\:s}$ and $\:{E}_{\varphi\:s}$ For the mth and the sth element of the MIMO system. The relation between the correlation coefficient_MBA_ and the ECC_MBA_ in the fading Rayleigh channel is given by^48^

24 $$\:{\rho\:}_{e}={\left|{\rho\:}_{c}\right|}^{2}$$

The S-parameter evaluation/calculation of ECC_**DWA**_ is given for the mth and sth ports by^48^

25 $$\:{ECC}_{\text{D}\text{W}\text{A}}={\rho\:}_{e}\left(m,s,N\right)=\frac{{\left|\sum\:_{n=1}^{N}{S}_{m,n}^{*}\:{S}_{n,s}\right|}^{2}}{{\pi\:}_{k=(m,s)}\left[1-\sum\:_{n=1}^{N}{S}_{m,n}^{*}\:{S}_{n,k}\right]}$$

Here, for the N-element system (n×n) symmetrical MIMO_DWA_-system, $\:{\rho\:}_{e}$ (m.s.n) corresponds to ECC_DWA_ between the mth and sth port. The expression of Eq. (25) is rewritten as

26 $$\:{ECC}_{DWA}=\frac{{\left|{S}_{mm}^{*}{S}_{ms}+{S}_{sm}^{*}{S}_{ss}\right|}^{2}}{\left(1-{\left|{S}_{ii}\right|}^{2}-{\left|{S}_{sm}\right|}^{2}\right)\:\left(1-{\left|{S}_{ss}\right|}^{2}-{\left|{S}_{ms}\right|}^{2}\right)}$$

Considering the two-port network (m = 1,2, s = 1,2) and four-port network (m = 1,2,3,4, s = 1,2,3,4), the following Equations evaluate ECC_MBA_ for ECC_two−port_ and ECC_four−port_ by

27 $$\:{ECC}_{DWA\left(Two\:Port\right)}=\frac{{\left|{S}_{11}^{*}{S}_{12}+{S}_{12}^{*}{S}_{22}\right|}^{2}}{\begin{array}{c}\left(1-{\left|{S}_{11}\right|}^{2}-{\left|{S}_{21}\right|}^{2}\right)\\\:\:\left(1-{\left|{S}_{12}\right|}^{2}-{\left|{S}_{22}\right|}^{2}\right)\end{array}}$$

28 $$\:{ECC}_{DWA\left(Four\:Port\right)}=\frac{{\left|{S}_{11}^{*}{S}_{12}+{S}_{12}^{*}{S}_{22}{+S}_{13}^{*}{S}_{32}+{S}_{14}^{*}{S}_{42}\right|}^{2}}{\begin{array}{c}\left(1-{\left|{S}_{11}\right|}^{2}-{\left|{S}_{21}\right|}^{2}-{\left|{S}_{31}\right|}^{2}-{\left|{S}_{41}\right|}^{2}\right)\\\:\:\left(1-{\left|{S}_{12}\right|}^{2}-{\left|{S}_{22}\right|}^{2}-{\left|{S}_{32}\right|}^{2}-{\left|{S}_{42}\right|}^{2}\right)\end{array}}$$

As already mentioned, the ECC_DWA_ value must be 0, which is an ideal case with the radiating elements achieving infinite isolation. However, the ideal case practically is not achievable, but the values of ECC_DWA_ within the operating bandwidth should be $\:\le\:$0.50. Figure 7a, b show the plot of ECC_DWA_ between port1_DWA_-port2_DWA_, port1_DWA_-port3_DWA,_ and port1_DWA_-port4_DWA_ for both methods, using S-parameter and by 3D-radiation. The simulated maximum values of ECC_DWA_ in Band-A correspond to less than 0.1, and for Band-B, the values are less than 0.009, as noted from Fig. 7a. The measured values of ECC_MBA_ are also plotted in Fig. 7a with a maximum value of 0.07 for Band-A and 0.03 for Band-B. For both the bands, Band-A and Band-B, the simulated and measured values are much lower than the ideal values, suggesting very little interference of the radiating fields between any two-port comparison. Figure 7(b) represents the simulated and measured ECC_DWA_ by the 3D-radiation method and uses Eq. (18) to evaluate the values. The values of simulated and measured ECC_DWA_ are below 0.20 and are more compared with ECC_DWA_ achieved by S-parameter, due to losses also being added in the latter case.

The parameter Diversity-Gain_DWA_ (DG_DWA_) signifies the merit of the signal reliability, which is due to the spatial diversity employed in the current work. It also measures the fading effects reduction, which is achieved by transmitting and receiving identical multiple copies over the independent channel path. The strong reliability of the signal is achieved by ensuring the reception of at least one of the transmitted signals. The DG_DWA_ is correlated with ECC_DWA_ by following the equation given below^48^.

29 $$\:{DG}_{DWA}=10\sqrt{1-{\left|{\rho\:}_{e}\right|}^{2}}$$

The DG_DWA_ values, as per the standard values, are $\:{DG}_{DWA}\ge\:9.95dB$. The simulated values of DG_DWA_ in Band-A and Band-B are more than 9.95dB, noted in Fig. 7c. Figure 7c also records the measured values that are greater than 9.6dB in Band-A and more than 9.7dB in Band-B.

The reflection performance and the matching of the impedance are measured by the MIMO parameter known as Total-Active-Reflection-Coefficient_DWA_ (TARC_DWA_), and also infer the amount of power reflected by the antenna in MIMO_MBA_. It can be stated that it is the ratio of the net reflected power to the net incident power between the two ports. The calculation of TARC_DWA_ also includes all the active ports working between the multiple antennae, which also includes mutual coupling. TARC_DWA_ calculation plays a vital role in the evaluation of the MIMO_DWA_-antenna networks and is a frequency-dependent parameter that changes with phase excitation. The power related to TARC_DWA_ is expressed as^48^

30 $$\:{\varGamma\:}_{a}^{t}=\frac{Available\:Power\:\left(AP\right)-Radiated\:Power\:\left(RP\right)}{Available\:Power\:\left(AP\right)}$$

For a lossless MIMO_DWA_ system with an N-number of elements or N ports, the TARC_DWA_ is given by^48^

31 $$\:{\varGamma\:}_{a}^{t}=\frac{\sqrt{\sum\:_{i=1}^{N}{\left|{b}_{i}\right|}^{2}}}{\sqrt{\sum\:_{i=1}^{N}{\left|{a}_{i}\right|}^{2}}}$$

Where [b]=[s][a]; a is the incident power with random phase, and b is the reflected power.

32 $$\:{b}_{1}={S}_{11}{a}_{1}+{S}_{12}{a}_{2}={S}_{11}{a}_{0}{e}^{j{\theta\:}_{1}}+{S}_{12}{a}_{0}{e}^{j{\theta\:}_{2}}={a}_{1}\left({S}_{11}+{S}_{12}{e}^{j\theta\:}\right)$$

33 $$\:{b}_{2}={S}_{21}{a}_{1}+{S}_{22}{a}_{2}={S}_{21}{a}_{0}{e}^{j{\theta\:}_{1}}+{S}_{22}{a}_{0}{e}^{j{\theta\:}_{2}}={a}_{1}\left({S}_{21}+{S}_{22}{e}^{j\theta\:}\right)$$

Combining Equations (25) and Eq. (26), TARC_MBA_

34 $$\:{\varGamma\:}_{a}^{t}=\frac{\sqrt{{\left|{S}_{11}+{S}_{12}{e}^{j\theta\:}\right|}^{2}+{\left|{S}_{21}+{S}_{22}{e}^{j\theta\:}\right|}^{2}}}{\sqrt{2}}$$

The simulated values of TARC_DWA_ both in Band-A and Band-B are more than 4.0dB as recorded in Fig. 7d, while the measured values are also plotted in Fig. 7d, and in both bands are greater than 2.5dB, which is above the condition of TARC_DWA_$\:\le\:$0.0dB.

The mismatch between the impedance of the antenna not only degrades the capacity but also includes factors such as mutual coupling and correlation. The Channel-Capacity-Loss_DWA_ (CCL_DWA_) also measures the availability of the capacity channel when compared with the uncorrelated and is measured by b/s/Hz. Typically, the CCL_DWA_ is written as^48^

35 $$\:{CCL}_{DWA}={log}_{2}\left(\text{det}\left({I}_{a\times\:a}+\frac{\rho\:}{{A}_{t}}A\right)\right)-{log}_{2}\left(\text{det}\left({I}_{a\times\:a}+\frac{\rho\:}{{A}_{t}}{A}_{ideal}\right)\right)$$

where, $\:{I}_{a\times\:a}$ Is the identity-matrix, ρ-SNR, A_t_- no. of transmitter antennas, A_ideal_ is the MIMO_DWA_ channel correlation-matrix.

The maximum transmission rate of the information over the channel with maximum limit is defined by Channel-Capacity-Loss_DWA_ (CCL_DWA_) with $\:{CCL}_{DWA}\le\:0.40b/s/Hz$. The CCL_DWA_ for a four-port MIMO_DWA_ antenna in generalized form is calculated by^48^

36 $$\:{CCL}_{DWA}=-{log}_{2}det\left({\alpha\:}^{s}\right)$$

where

37 $$\:{\rho\:}_{mm}=1-{\sum\:}_{n=1}^{4}{\left|{S}_{mn}\right|}^{2}$$

38 $$\:{\rho\:}_{ms}=-\left({S}_{mm}^{*}{S}_{ms}+{S}_{sm}^{*}{S}_{ms}\right)$$

Figure 7e shows the calculation of CCL_MBA_ for both simulated and measured reflection and transmission coefficients. The simulated average values $\:{CCL}_{DWA}\le\:0.25b/s/Hz,$ and the measured averaged values correspond to $\:{CCL}_{DWA}\le\:0.21b/s/Hz$.

Mean-Effective-Gain_MBA_(MEG_DWA_) quantifies the ability of the MIMO_DWA_ antenna to receive power efficiently in a multipath fading channel.

In general, the MEG_ms_ are calculated by^48^

39 $$\:{MEG}_{ms}=0.5\left[1-\sum\:_{s=1}^{K}{\left|{S}_{ms}\right|}^{2}\right]$$

40 $$\:{MEG}_{m}=0.5\times\:(1-{\left|{S}_{mm}\right|}^{2}-{\left|{S}_{ms}\right|}^{2})$$

41 $$\:{MEG}_{s}=0.5\times\:(1-{\left|{S}_{ss}\right|}^{2}-{\left|{S}_{sm}\right|}^{2})$$

The ratio calculates the MEG_DWA,_ which is given by^48^

42 $$\:\frac{{MEG}_{m}}{{MEG}_{s}}=\frac{0.5\times\:(1-{\left|{S}_{ms}\right|}^{2}-{\left|{S}_{ms}\right|}^{2})}{0.5\times\:(1-{\left|{S}_{ss}\right|}^{2}-{\left|{S}_{sm}\right|}^{2})}$$

**Fig. 8:**
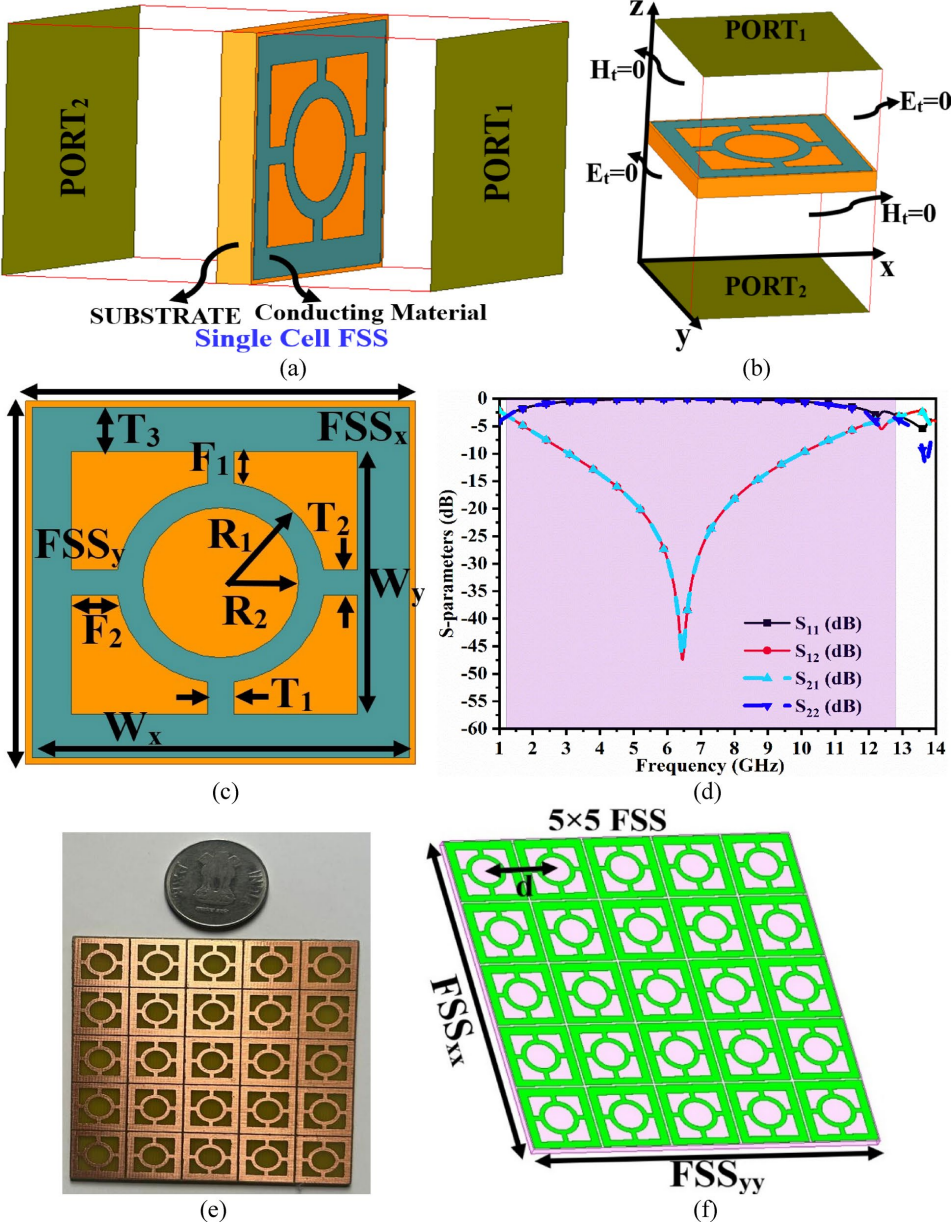

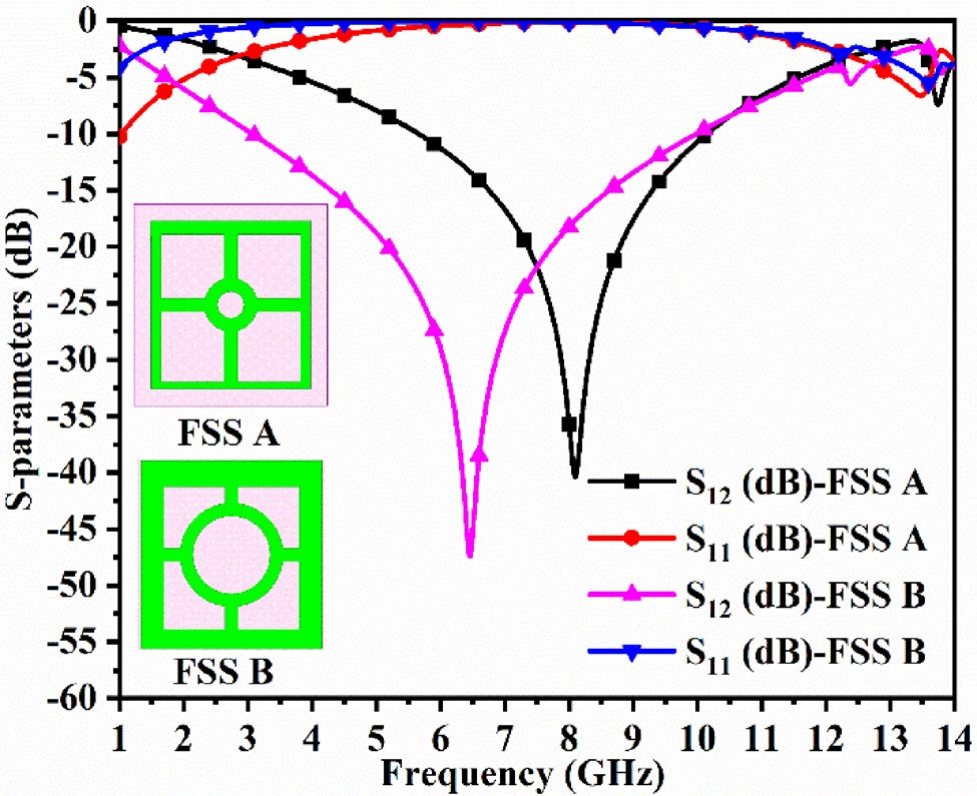
Single/Array FSS_DWA_ (**a**) 3-D model with port excitation (**b**) 3-D model with boundary conditions (**c**) Optimal dimensions (**d**) S-parameter results (**e**) 5 × 5 FSS_DWA_ array (**f**) Evolution of array-FSS_DWA_.

For perfect balance, the MEG_DWA_ between the two antenna ratios should be 1 (0dB). The proposed antenna MEG_MBA_ is plotted in Fig. 8 with simulated MEG_DWA_ corresponding to Fig. 7f,g,h maintains a ratio of 0.0dB. The measured MEG_DWA_ are plotted in Fig. 7f, g, h, which also maintain the ratio between the two MEG_MBA_ plot ratios of 0.0dB. Table 5 consolidates the comparison of all the diversity parameters obtained from simulation and measured results, with the values in both cases well below the threshold values.

## Single/Array FSS_DWA_

The Frequency-Selective-Surface, also known as meta-materials or meta-surfaces, is are artificially engineered repetitive structure designed to serve applications either to block or allow electromagnetic waves by transmitting and reflecting them. The shape of the periodic structure can be cross-monopole, loops, hexagonal, convoluted-shaped, circular-ring, hexagonal-ring, etc. The unit cell is defined as the element that produces the frequency response and is determined by its geometry. The FSS_**DWA**,_ working as the filter, is divided into four categories, namely low-pass, high-pass, band-stop, and bandpass filters.

In the proposed work, two measured bands of the four-port MIMO_**DWA**_ antenna, designated as Band-A and Band-B, achieve − 10.0dB impedance bandwidth of 4.04–6.64 GHz and 7.44–16.60 GHz. The objective of the work also includes enhancing the gain of the antenna in the above-measured bandwidths, and hence, the new additional technique needs to be explored. The frequency-selective surface is one of the options to increase the gain, and hence, the FSS is designed which cover the bandwidth desired by the MIMO_**MBA**_ antenna and is integrated with the proposed MIMO_**DWA**_ antenna. However, initially, the FSS_**DWA**_ needs to be characterized with boundary conditions, including port excitation with the EM wave that needs to be incident from PORT1, and needs to verify the transmitted signal reaching PORT2 and reflected at PORT1.

The other pair of boundary faces is assigned as Et = 0 boundary condition, and the remaining pair of faces is assigned as H_t_=0 boundary condition, as shown in Fig. 8b. The detailed optimal dimensions of the proposed single-element FSS are shown in Fig. 8c with the corresponding S-parameter plotted in Fig. 8d concerning PORT1 and PORT2. The overall dimension of the single-element FSS corresponds to FSS_x_×FSS_y_=14.50 × 15.0 mm^2^. The metallic structure is printed on the top surface of the FR4 dielectric with ε_r_ = 4.40. The metallic structure consists of a rectangular ring with an outer dimension of W_x_=14.0 mm and an inner dimension of W_y_=14.50 mm. The thickness of the rectangular ring corresponds to the dimension of T_3_ = 2.00 mm. The FSS also includes a circular ring placed at the center as shown in Fig. 8c with an outer-ring radius of R_1_ = 4.00 mm and an inner-ring radius of R_2_ = 3.00 mm. The rectangular-ring and the circular-ring are connected with four arms, which are identical in length and breadth of dimension F_1_ = F_2_ = 1.28 mm and T_1_ = T_2_ = 1.00 mm. This forms the complete proposed FSS_**DWA**_ structure with simulated S-parameters plotted in Fig. 8d. The reflection coefficients, S_11_/S_22_ (dB), are below − 3.0dB with a bandwidth corresponding to 1.23–12.20 GHz. This implies that reflection-coefficients S_11_/S_22_=$\:20log\frac{{V}_{reflected}}{{V}_{incident}}$ They are reflected from the surface of the metallic structure of the designed FSS. On the other hand, the transmission coefficient, S_12_/S_21_=$\:20log\frac{{V}_{transmitted}}{{V}_{incident}}$ It is more than 5.0dB and deepens for the bandwidth between 4.00 and 12.0 GHz, indicating very little power passing through the FSS. This also indicates that maximum power is reflected through the FSS from PORT1 to PORT2 and behaves as a band-stop filter for the above-mentioned bandwidth. Hence, Fig. 8 concludes the single-element FSS suitable for wideband applications with band-stop characteristics suitable for integration with an antenna working in the same bandwidth.

**Fig. 9:**
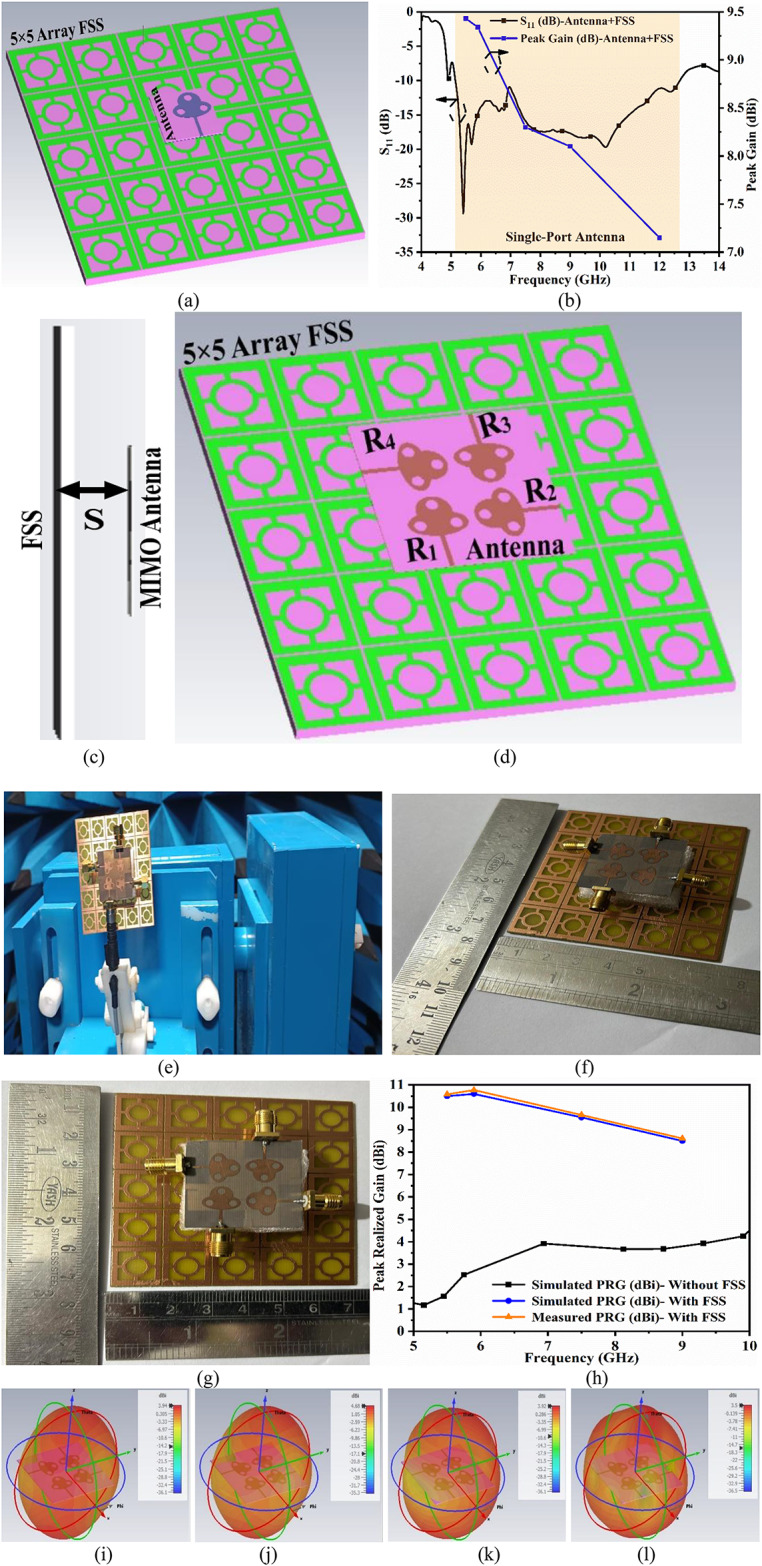

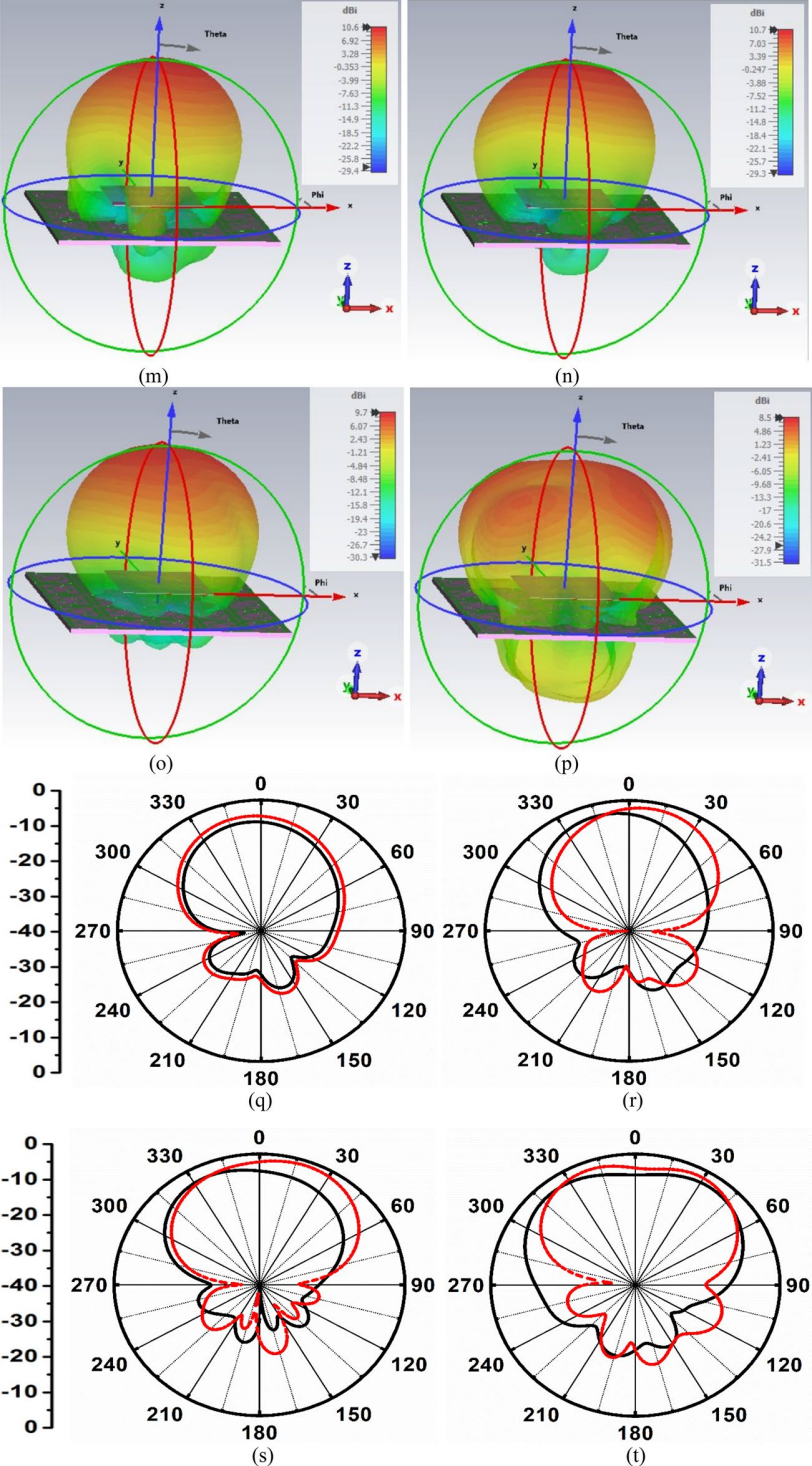
Integration of FSS_DWA_ with proposed antenna (**a**) Single-port antenna placed above FSS dual-band array (**b**) S-parameter and peak realized gain plot (**c**) Side-view of Antenna and FSS (**d**) Proposed MIMO antenna placed above FSS-array (**e**) Fabricated MIMO-FSS antenna placed within Anechoic Chamber (**f**) Top-view of fabricated Antenna integrated with FSS_DWA_-array (**g**) 3D-view of fabricated Antenna integrated with FSS_DWA_-array (**h**) Simulated-Measured PRG (without and with FSS_DWA_-array); 3D-radiation of MIMO antenna without FSS at (**i**) 5.50 GHz (**j**) 5.90 GHz (**k**) 7.50 GHz (**l**) 9.90 GHz; 3Dradiation patterns with loaded FSS at (**m**) 5.50 GHz (**n**) 5.90 GHz (**o**) 7.50 GHz (**p**) 9.90 GHz; Simulated-Measured 2D-radiation patterns at (**q**) 5.50 GHz (**r**) 5.90 GHz (**s**) 7.50 GHz (**t**) 9.90 GHz.

Figure 8 also shows the repetitive structure of the extended single-element FSS shown in Fig. 8c. The FSS-array has a dimension of FSS_xx_×FSS_yy_=72.5 × 75 mm^2^ with center-to-center adjacent single element FSS_**DWA**_-structure of d = 15.0 mm as shown in Fig. 8e. The FSS_**MBA**_ array also achieves the identical S-parameter results plotted in Fig. 9d because it is the extension of a single-element FSS_**MBA**_. Figure 8f records the S-parameter results for two different dimension FSS_**MBA**_ structures marked as FSS-A and FSS-B. The dimension of the FSS-A is reduced, with the bandwidth also shifting and deviating from the required wideband bandwidth. However, FSS-B is the proposed FSS_**DWA**_ structure, which produces the required wideband bandwidth with minimum transmitted through FSS_**DWA**_ and maximum reflected signal from the metallic surface of FSS_**DWA**_ as noted from Fig. 8f.

## Single-Port/Four-Port MIMO antenna loaded with FSS_DWA_

As already mentioned earlier, the objective of the work is to design an antenna with integrated FSS_**DWA**_ for the enhancement of peak realized gain (PRG). Figure 9 gives more insight into the integration of FSS_**DWA**_ with the antenna for single-port and MIMO_**DWA**_-configuration of the antenna with FSS_**DWA**_-array. Figure 9a shows the integration of a single-port antenna placed over the 5 × 5 FSS_**DWA**_ array. The distance between the array and the antenna corresponds to a distance of **S**, with a value of $\:S\le\:\frac{{\lambda\:}_{c}}{4}$ where λ_c_ is the lower cut-off frequency (5.0 GHz) of the operating bandwidth, and the distance of λ_c_ = 15.0 mm is maintained between the antenna and the FSS for maximum reflection at the lower cut-off frequency of the operating bandwidth. This distance ensures maximum reflection from the surface of FSS_**DWA**_ when the antenna is excited.

**Table 6:** Maximum peak realized gain for a single-port antenna with loaded FSS.

Frequency (GHz)	Maximum Peak Realized Gain (dBi) Main Lobe	Maximum Radiation Level of Back Lobe (dBi)
5.50	9.43	-21.70
5.90	9.34	-24.20
7.50	8.30	-25.20
9.00	8.10	-17.20
12.0	7.15	-8.60

**Table 7:** Maximum peak realized gain for a Single-port antenna with a loaded FSS.

Frequency (GHz)	Simulated		Measured	
	Maximum Peak Realized Gain (dBi) Main Lobe	Maximum Radiation Level of Back Lobe (dBi)	Maximum Peak Realized Gain (dBi) Main Lobe	Maximum Radiation Level of Back Lobe (dBi)
5.50	10.50	-19.50	10.58	-19.63
5.90	10.60	-21.80	10.77	-21.89
7.50	9.55	-23.40	9.66	-24.32
9.00	8.50	-18.12	8.61	-18.23

The S-parameter result, S_11,_ and peak-realized gain are plotted in Fig. 9b with the − 10.0dB bandwidth corresponding to 5.14–12.68 GHz. The peak-realized-gain are calculated 5.50 GHz, 5.90 GHz, 7.50 GHz, 9.00 GHz and 12.0GHzwith corresponding peak-realized-gain of 9.43dBi, 9.34dBi, 8.30dBi, 8.10dBi, 7.15dBi respectively. It can be observed that the antenna is highly directive with minimal back lobe radiation, and increases the peak realized gain. Table 6 shows the values of peak realized gain for frequencies corresponding to applications including WLAN, V2X, Uplink/Downlink Satellite systems, Ultra-wideband imaging and surveillance systems, RADAR, and FR1 band applications. Figure 9c shows the side-view of the integrated FSS with antenna, and the distance of **S** is marked for maximum reflection, and Fig. 9d shows the 3D-view of the MIMO-FSS Array simulation model. Figure 9e is the photograph of the hardware (MIMO antenna and FSS) placed on the rotor within the anechoic chamber for far-field results measurement for Peak-Realized-Gain and 2D-radiation patterns. Figure 9f and Fig. 9g signify the proposed four-port MIMO_**DWA**_ antenna placed over the proposed 5 × 5 FSS_**DWA**_-array structure. The impact of the FSS_**DWA**_-array over the antenna is to increase the peak realized gain, which is shown in Fig. 9h. The peak-realized gain in the absence of the FSS_**DWA**_ achieves a maximum value of 5.20dBi at 11.10 GHz.

Figure 9i-l represents the 3D radiation patterns of the four-port MIMO antenna without loading of FSS at 5.50 GHz, 5.90 GHz, 7.50 GHz, and 9.0 GHz. The antenna offers conventional 3D-radiation patterns with a donut shape corresponding to dipole-like and omnidirectional patterns in the yz-plane and xy-plane. Figure 9m-p demonstrates the significance of loading the proposed FSS_**DWA**_ with the proposed four-port MIMO antenna. The four 3D-radiation patterns are plotted for frequencies corresponding to 5.50 GHz, 5.90 GHz, 7.50 GHz, and 9.0 GHz, respectively. The significant suppression of the back lobes is observed from all three 3D-radiation patterns, with maximum peak-realized-gain recorded at 5.90 GHz. This also concludes that with the optimized distance of 15.0 mm, the proposed FSS shows the ability to reflect the back lobes significantly for all four application frequency values, and concludes that the FSS_**DWA**_ blocks the back radiation, which confirms the band-stop characteristics. Figure 9q-t represents the comparison of the simulated-measured 2D-plot in the boresight direction. The simulated and measured 2D-radiation patterns are in good agreement with each other, and the comparison is tabulated in Table 7. The simulated peak-realized-gain varies between 8.50dBi-10.60dBi, and the measured peak-realized-gain varies between 8.61dBi-10.58dBi. The gain of the antenna is enhanced by the integration of FSS_**DWA**,_ and the objective of loading the wideband FSS_**DWA**_ is achieved.

The discrepancy between the simulated and the measured results is due to the error in the accurate fabrication process and respective measurement, which requires accuracy in calibration of the VNA before measurement. The connectors soldered which are used in the MIMO system for feeding the antenna also need to be soldered accurately with proper grounding.

**Fig. 10:**
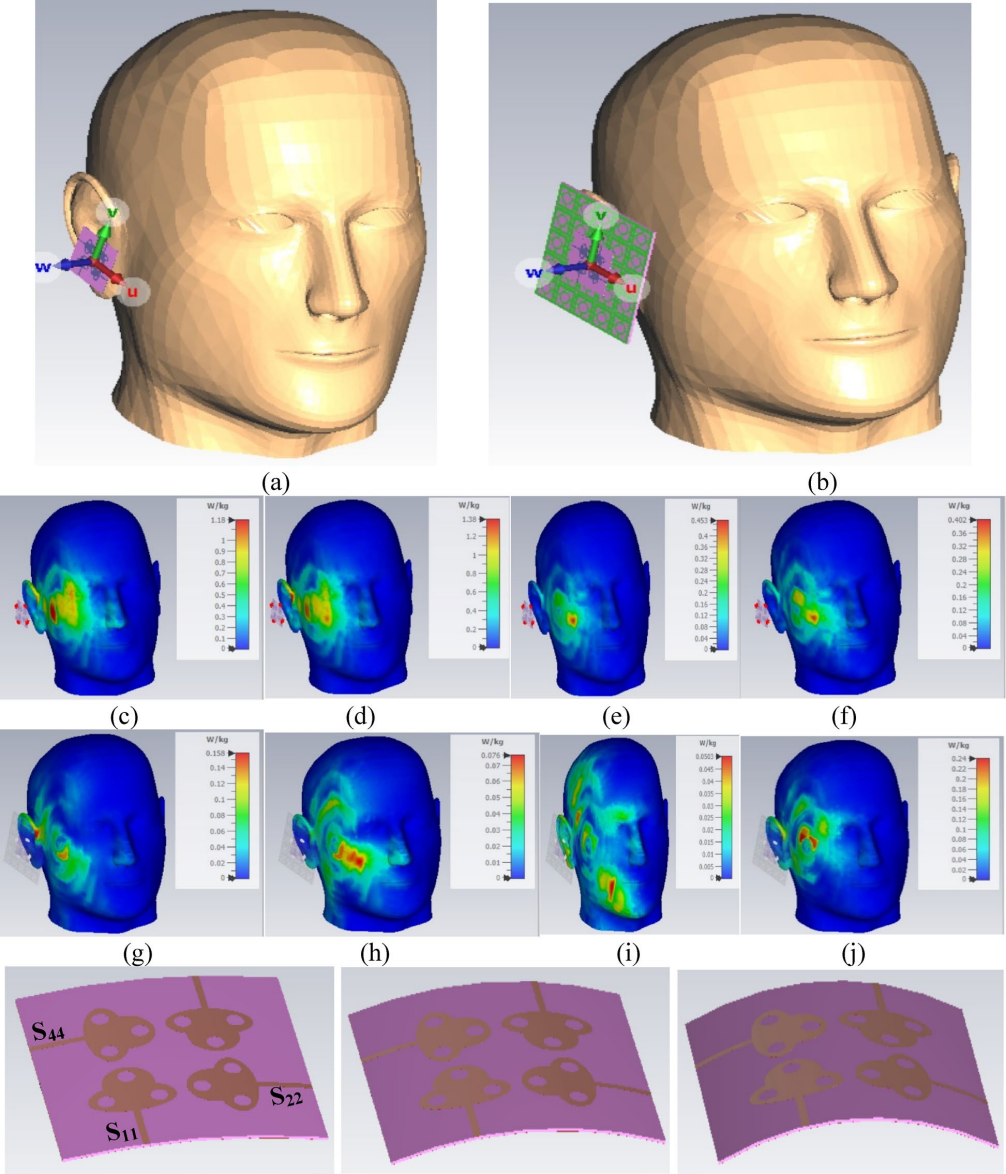

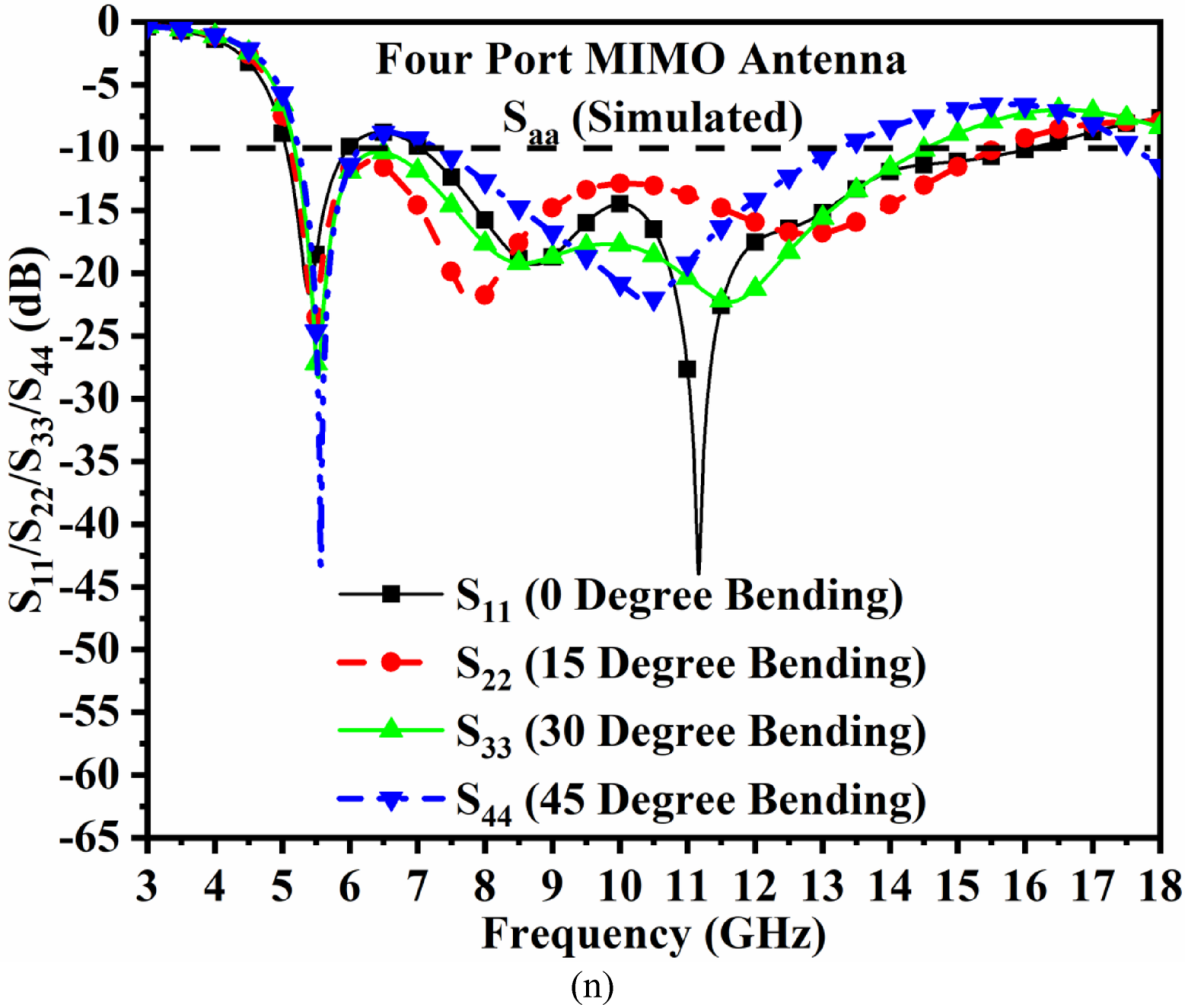
SAR_DWA_ Analysis (**a**) MIMO antenna placed near head-phantom without FSS (**b**) MIMO_DWA_ antenna integrated with FSS_DWA_ placed near head-phantom; SAR analysis without FSS of MIMO antenna at (**c**) 5.50 GHz (d) 5.90 GHz (**e**) 7.50 GHz (**f**) 10.0 GHz; SAR analysis with integrated FSS of MIMO antenna at (**g**) 5.50 GHz (**h**) 5.90 GHz (**i**) 7.50 GHz (**j**) 10.0 GHz; Bending Analysis of MIMO antenna at angles of (**k**) 15^◦^ (**l**) 30^◦^ (**m**) 45^◦^ (**n**) S-parameter of bending angles.

As the proposed four-port MIMO_**DWA**_ antenna is suitable for lower frequency bands, including WLAN, V2X, Uplink/Downlink Satellite systems, Ultra-wideband imaging and surveillance systems, RADAR, and FR1 band applications, the antenna can also be used for IoT and Biomedical applications. Hence, the two vital analyses, known as SAR_**MBA**_ and the bending of the antenna at different angles, need to be studied. Figure 10a and b show the use of a human phantom model with 1 g of the tissue model, including skin, fat, and muscle, considered in the simulation. Figure 10a corresponds to the MIMO antenna placed near the human-phantom model, and Fig. 10b shows the integrated FSS-MIMO antenna also placed near the human phantom by a distance of 5.0 mm in both scenarios. Figure 10c, Fig. 10d, Fig. 10e and Fig. 10f corresponds to the SAR_**MBA**_ simulation at frequency values of 3.50 GHz, 5.90 GHz, 7.50 GHz and 10.0 GHz. Specific-Absorption-Rate (SAR) is defined as the amount of radiated power absorbed per kg of the human body and is calculated by Eq. (42)^48^

42 $$\:{SAR}_{n-s}=\frac{\sigma\:{\left|E\right|}^{2}}{\rho\:}$$

σ-conductivity of body tissue (S/m).

E-applied electric field (V/m).

The ρ-mass density of the body tissue (kg/m^³^).

As per Eq. (42), SAR is calculated based on the power absorbed by the body and is directly proportional to the conductivity of the body tissue (σ-S/m; skin, fat, muscle), the magnitude of the incident electric field intensity ($\left\lceil E \right\rceil$ – V/m) and inversely proportional to the mass dvalues of the net impedanceensity of the tissue (Kg/m^3^). Thus, the equation suggests that the tissue model needs to be considered, comprising the three layers, namely skin, fat, and muscle. The interaction of the EM wave with the tissue model is given by SAR_**DWA**,_ whose values, as per the IEEE standards, must be SAR_**DWA**_$\:\le\:$1.60 W/Kg for 1 g of the tissue. Hence, the electrical properties of the tissue model, including electrical permittivity, conductivity, and loss tangent, need to be known^52,^^53^, which are tabulated in Table 8. Figure 10c, Fig. 10d, Fig. 10e, and Fig. 10d show the simulation-calculation of SAR_**DWA**_ for a four-port MIMO_**DWA**_ antenna without inclusion of FSS at frequency values of 5.50 GHz, 5.90 GHz, 7.50 GHz, and 10.60 GHz, respectively. The minimum value of SAR_**DWA**_ corresponds to 0.402 W/Kg at 10.0 GHz and 1.38 W/Kg at 5.90 GHz. The comparison of SAR at selected frequency values without and with FSS is tabulated in Table 9. Also, with the integration of FSS_**DWA**_ with the MIMO_**DWA**_ antenna, the maximum SAR_**MBA**_ values correspond to 0.158 W/Kg at 5.50 GHz. The SAR_**DWA**_ values are reduced for the FSS integrated antenna as recorded in Fig. 10g, h, i, and j, which concludes that the FSS_**DWA**_ reduces the interaction of the EM-wave with the human-phantom, which reduces SAR_**DWA**_ because the electric field intensity is also reduced. This ensures that the proposed MIMO_**DWA**_ antenna can be used in the application for MIMO_**DWA**_ antenna without and with FSS_**DWA**,_ due to the reasons that in both cases, the SAR_**DWA**_ values are well below the specified value ($\:\le\:1.60\:W/kg)$. Hence, the proposed MIMO antenna loaded with FSS is in compliance with the FCC with a limit of 1.60 W/kg for an average 1 g of tissue with 50 mW power of less than 2.0 W/kg as per the ICNIRP standard for an average of 10 g tissue.

**Table 8:** Tissue model EM properties^52,^^53^.

Tissue	Frequency (GHz)	Electrical permittivity	Conductivity	Loss Tangent	Density (Kg/m^3^)
Skin	5.50	35.4	3.46	0.32006	1109
	5.90	35.0	3.80	0.33082	
	7.50	33.6	5.31	0.0067	
	10.0	31.3	8.02	0.46038	
Fat	5.50	9.94	0.774	0.17956	911
	5.90	9.83	0.852	0.1846	
	7.50	9.41	1.18	0.20356	
	10.0	8.80	17.1	0.22857	
Muscle	5.50	48.9	4.61	0.30815	1090
	5.90	48.4	5.08	0.32017	
	7.50	46.2	7.12	0.006	
	10.0	42.8	10.6	0.44666	

**Table 9:** SAR values (without and with FSS).

Sl. No.	Frequency (GHz)	SAR (W/Kg) Without FSS	SAR (W/Kg) With FSS	Ideal Values
1	5.50	1.18	0.158	1.60 W/Kg
2	5.90	1.38	0.076	
3	7.50	0.453	0.0503	
4	10.0	0.402	0.24	

Figure 10 also includes the conformal configuration of the MIMO_**DWA**_ antenna, which is printed on the thin Rogers substrate with a thickness of 0.254 mm. The bending of the antenna is subjected to 0◦ (no bending), 15^◦^ (*r* = 114 mm), 30^◦^ (*r* = 57.3 mm), and 45^◦^ (*r* = 38.2 mm) as shown in Fig. 10k, Fig. 10l, and Fig. 10m in the y-axis, and the corresponding S-parameters are plotted in Fig. 10n. Figure 10n concludes that there is not much impact on the operational-measured bandwidth of the MIMO_**DWA**_ antenna other than the reduction of the higher cut-off frequency, but includes all the applications, including WLAN, V2X, UWB, Uplink/Downlink Satellite system, RADAR, and FR1 bands.

## State-of-the-Art comparison

**Table 10:** Performance comparison.

Ref	Size of the Antenna (mm^2^)/ λ_o_^2^	Bandwidth (GHz)	No. of Ports	Isolation (dB)	ECC	DG (dB)	TARC (dB)	CCL (b/s/Hz)	MEG port_A_-port_B_
^1^	48 × 34 0.93λ_o_ × 0.66λ_o_	3.52–10.08	4	< 23.01	< 0.039	> 9.59	<-15.0	< 0.29	$\:\cong\:$0.0
^2^	73.3 × 73.3 1.32λ_o_ × 1.32λ_o_	3.3-13.84	4	< 10.0	0.20	NC	< 14.0	NC	NC
^8^	64 × 64 0.72λ_o_ × 0.72λ_o_	2.08–10.4	4	< 15.0	0.10	> 9.90		< 0.30	$\:\cong\:$0.0
^12^	45 × 45 0.76λ_o_ × 0.76λ_o_	3.1–13.1	4	< 17.10	0.02	> 9.9985	< 25.0	NC	NC
^17^	66 × 33 0.58λ_o_ × 0.29λ_o_	2.1–2.95 5.1–5.90	2	< 27.0	0.20	> 9.99	NC	< 0.23	$\:\cong\:$0.0
^19^	20 × 34 0.27λ_o_ × 0.46λ_o_	2.46–13.98	2	< 10.0	0.003	> 9.96	< 10.0	< 0.30	$\:\cong\:$0.0
^30^	58.45 × 58.45 4.31λ_o_ × 4.31λ_o_	13.6–17.50	4	< 30.01	0.001	NC	< 10.0	< 0.11	NC
^27^	32 × 32 0.56λ_o_ × 0.56λ_o_	3.20–5.20	4	< 25.0	0.11	NC	NC	NC	NC
*P	30 × 30 0.51λ_o_ × 0.51λ_o_	4.04–6.64 7.44–16.60	4	< 10.0	0.01 0.07	> 9.70 > 9.85	< 5.0 < 2.5	< 0.38 < 0.30	$\:\cong\:$0.0

**Table 11:** Performance analysis comparison.

Ref	FSS Integration (Y/*N*)	Peak Realized Gain (dBi)	SAR Analysis	Conformal Analysis	CMA Analysis
^1^	N	2.91	N	N	N
^2^	N	6.0	N	N	Yes 10-modes
^8^	Y	9.57	N	N	N
^12^	N	4.00	N	N	N
^17^	N	6.25	Y	N	N
^19^	N	5.71	N	N	N
^30^	N	6.05	N	N	N
^27^	N	4.01	N	N	N
*P	Y	10.77	Y	Y	Yes 10-modes

**Table 12:** Comparison of the hybrid antenna with other published work.

Ref	Antenna Size (mm^2^)	Gain (dBi)	Isolation (dB)	Applications
^16^	20.0 × 24.0	5.12	> 15.0	Uplink/Downlink (7.25 GHz- 8.320 GHz) X-Band, Ku-Band, ISM Band (24.0 GHz), 24.0 GHz UWB Band, n258, n257/n261, n262, n263
^25^	18.0 × 8.50	7.73	> 20.0	n257/n261 n260
^26^	40.0 × 40.0	5.0	> 20.5	WLAN, C-band Uplink, GEOSAT LNB, ITU
^31^	56.0 × 56.0	3.25	> 20.0	Chinese 5G, n78, n79, WiFi 6E
^35^	31.0 × 42.0	7.125	> 30.0	n260, n261
*P	30.0 × 30.0	10.77	> 10.0	Sub-6 GHz 5G, WLAN, X-band, Satellite communication

The proposed four-port FSS-integrated antenna is compared with state-of-the-art similar published work tabulated in Tables 10 and 11, respectively. The comparison shows that the antenna is compact, with good diversity parameters achieved. Also, it can be seen that all the analyses, including SAR_**DWA**_, Conformal_**DWA**,_ and CMA_**DWA**_ analyses, are applied to the proposed work, with either one or none of the analyses seen in other published work. The FSS integration of the MIMO_**DWA**_ antenna achieves a maximum peak-realized gain of 12.68dBi. The proposed MIMO_**DWA**_ integrated with FSS_**DWA**_ is suitable for multiple wireless applications, with bending analysis suggesting integration of the antenna in the field of flexible electronics. Table 12 also shows the comparison of the hybrid proposed MIMO antenna with other published work, where the antenna achieves the highest peak-gain of 10.77 dBi in comparison with the compared work.

## Conclusions and future scope of work

In this work, a four-port MIMO_**MBA**_ antenna integrated with a novel wideband frequency-selective surface is investigated. The proposed antenna is fabricated on a Rogers 0.254 mm thickness substrate with a size of 30 mm×30 mm, and the FSS_**DWA**_ is printed on a FR-4 substrate with a size 72.5 mm×75.0 mm with a thickness of 1.60 mm. The Clubs-shaped patch antenna and modified rectangular ground achieve − 10.0dB bandwidth of 4.04–6.64 GHz and 7.44–16.60 GHz, respectively, which are useful for multi-band wireless applications. The integrated FSS-MIMO antenna also achieves an average peak-realized gain of 12.0dBi with stable 2D-radiation patterns at 5.50 GHz, 5.90 GHz, 7.50 GHz, and 9.0 GHz. The antenna bandwidth is also realized by applying CMA_**DWA**_ analysis and proposing a conceptual equivalent-circuit-model analysis. The SAR_**DWA**_ analysis is applied to the proposed MIMO_**DWA**_ antenna by placing it near the human phantom model with values within the permissible range, and it also achieves good diversity performance by calculating ECC_**DWA**_, DG_**DWA**_, TARC_**DWA**_, CCL_**DWA**,_ and MEG_**DWA**_. The conformal analysis performance suggests that the MIMO_**DWA**_ antenna can be useful in future flexible-electronics device integration, including displays.

The MIMO antenna can also be extended to an eight-port configuration, and can also be modified for an array configuration by using a matched corporate-feeding technique. The massive MIMO can be useful for 5G applications. The antenna can also be integrated with smart reconfigurable intelligent surfaces.

## Data Availability

The datasets used and/or analysed during the current study available from the first author (Manish Sharma) on reasonable request.
